# Fe(III)-Complex-Imprinted Polymers for the Green Oxidative Degradation of the Methyl Orange Dye Pollutant

**DOI:** 10.3390/polym13183127

**Published:** 2021-09-16

**Authors:** Paulina Haller, Ignacio Machado, Julia Torres, Agustina Vila, Nicolás Veiga

**Affiliations:** 1 Química Inorgánica, Departamento Estrella Campos, Facultad de Química, Universidad de la República (UdelaR), Av. Gral. Flores 2124, Montevideo 11800, Uruguay; paulina_hm@hotmail.com (P.H.); jtorres@fq.edu.uy (J.T.); 2 Química Analítica, Departamento Estrella Campos, Facultad de Química, Universidad de la República (UdelaR), Av. Gral. Flores 2124, Montevideo 11800, Uruguay; imachado@fq.edu.uy; 3 Laboratorio de Biocatálisis y Biotransformaciones, Departamento de Química Orgánica, Facultad de Química, Universidad de la República (UdelaR), Av. Gral. Flores 2124, Montevideo 11800, Uruguay; avila@fq.edu.uy

**Keywords:** molecular imprinting, Fenton-like catalysis, pollutant dye, methyl orange, green oxidative degradation

## Abstract

One of the biggest problems worldwide is the pollution of natural water bodies by dyes coming from effluents used in the textile industry. In the quest for novel effluent treatment alternatives, the aim of this work was to immobilize Fe(III) complexes in molecularly imprinted polymers (MIPs) to produce efficient Fenton-like heterogeneous catalysts for the green oxidative degradation of the methyl orange (MO) dye pollutant. Different metal complexes bearing commercial and low-cost ligands were assayed and their catalytic activity levels towards the discoloration of MO by H_2_O_2_ were assessed. The best candidates were Fe(III)-BMPA (BMPA = di-(2-picolyl)amine) and Fe(III)-NTP (NTP = 3,3′,3″-nitrilotripropionic acid), displaying above 70% MO degradation in 3 h. Fe(III)-BMPA caused the oxidative degradation through two first-order stages, related to the formation of BMPA-Fe-OOH and the generation of reactive oxygen species. Only the first of these stages was detected for Fe(III)-NTP. Both complexes were then employed to imprint catalytic cavities into MIPs. The polymers showed catalytic profiles that were highly dependent on the crosslinking agent employed, with N,N-methylenebisacrylamide (MBAA) being the crosslinker that rendered polymers with optimal oxidative performance (>95% conversion). The obtained ion-imprinted polymers constitute cheap and robust solid matrices, with the potential to be coupled to dye-containing effluent treatment systems with synchronous H_2_O_2_ injection.

## 1. Introduction

The world’s population is increasing rapidly and so is the need for consumer products. To keep up with that requirement, the global economy and industrial technology have rapidly developed, leading to a sustained increase in the pollution of ecosystems. Today, the quality of natural waters is in continuous decline, mainly due to the flourishing of industries that use dyes, such as those that produce fabrics, paper, cosmetics, plastics, rubber, leather, pharmaceuticals, and food [1,2]. Within this global context, huge amounts of polluting dyes are discharged into natural waters [3]. In China, for example, 1.6 trillion tons of waste containing dyes are dumped into watercourses each year [4]. In our country, Uruguay, dyes reach natural water reservoirs as well, mainly from the leather and textile industries [5,6].

Dyes are a major environmental issue. They are refractory to biodegradation and accumulate in the environment, polluting ecosystems [7,8]. In addition, they absorb light to a great extent, affecting the growth of phototrophic organisms, such as algae, plants, and other photosynthetic life forms. Additionally, the slow and long-term decomposition of these compounds gives rise to carcinogenic and mutagenic species, which affect the quality of water for human consumption [9,10,11].

**Figure 1:**
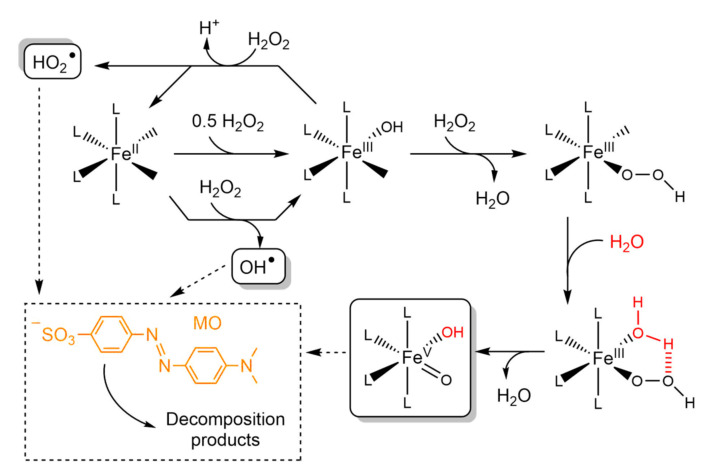
Mechanistic proposal for the catalytic activity of iron complexes towards the oxidation of organic compounds in the presence of H_2_O_2_. MO = methyl orange.

Azo dyes are synthetic organic compounds bearing one or several chromophore azo bonds (–N=N–) [12]. They account for up to 70% of the dyes in use nowadays, being widely employed in the food, pharmaceutical, cosmetic, textile, and leather industries [13]. An important member of this family is methyl orange (MO, Figure 1), which is mainly used as a pH indicator and dye for textiles [14]. MO is not biodegraded by sewage organisms [15], and its metabolites are toxic and mutagenic [16].

Recently, dye accumulation in the ecosystems has increased alarmingly, with the textile industry being responsible for the contamination of between 17 and 20% of natural water reservoirs [17,18]. To tackle this problem, a wide range of new methodologies for wastewater treatment have been developed, including adsorption, filtration, biological treatment, membrane separation, coagulation, flocculation, sonochemical degradation, electroreduction, and photolysis [8,19,20,21,22,23,24]; however, these strategies exhibit some disadvantages, such as incomplete decomposition of pollutants, increased solution density, high energy consumption, high operating costs, and excessive sludge formation [8]. Among these new alternative technologies, the advanced oxidation processes (AOPs) catalyzed by iron complexes have become promising approaches for the green and efficient oxidative degradation of pollutant dyes [8,25,26]. The use of Fe(II) and Fe(III) complexes as oxidation catalysts was initially exploited to achieve green and stereoselective oxidation of organic compounds in organic solvents [27,28,29,30]. From these works, it is now known that their catalytic activity in the presence of H_2_O_2_ depends on the structure of the metal complex and proceeds through an intricate mechanism involving the generation of reactive oxygen species (OH^●^, HO_2_^●^, etc.) and a highly reactive Fe(V) intermediate (Figure 1). Recently, some of these iron complexes and other coordination compounds have been tested as oxidation catalysts in water to promote the oxidative degradation of water-soluble organic pollutants under green conditions. Unfortunately, even though the results have been encouraging, the number of assayed metal complexes is still low [25,26,31,32].

One of the most important advances in this field has been the development of heterogeneous iron-containing catalysts [33,34,35], which can be removed and stored after operation, as well as being easily coupled to water treatment systems [8,33]. The catalysts’ supporting matrices used so far include carbon nanotubes with Fe_3_O_4_ [33], Fe_2_O_3_–carbon fiber [34], and iron complexes covalently bound to polymers [36], as well as complexing solid phases such as nafion, zeolite, activated charcoal, clay, resin, and silica [8]. Some of those solid phases, however, display medium-to-low conversions [13], have high costs, and involve complex synthetic procedures, since they are based on the design of tailor-made ligands or chemical grafting [36,37]. An interesting and unexplored alternative as a supporting phase is the use of molecularly imprinted polymers (MIPs) [38,39], since they can provide robust, inexpensive, and reusable matrix-bearing iron-loaded catalytic sites where the H_2_O_2_-mediated degradation of the target pollutants can occur [40]. In this regard, further research on Fenton-like catalysts based on iron-containing MIPs is needed as a part of the development of innovative and efficient technological solutions to the treatment of dye-containing industrial effluents [8].

**Figure 2:**
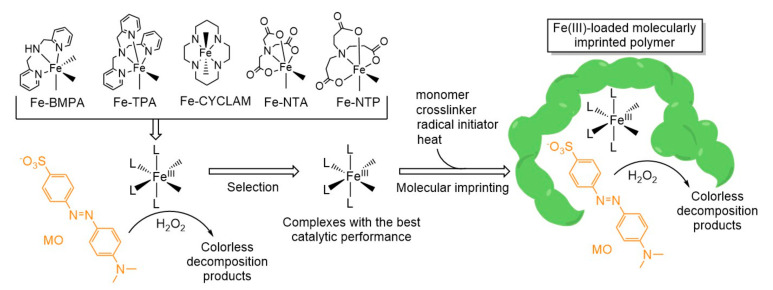
General strategy followed in this work.

In this work, we explored for the first time MIPs as supporting phases to develop new Fenton-like heterogeneous catalysts capable of promoting the green oxidative degradation of the azo dye methyl orange (Figure 1). As a first step, five different Fe(III) complexes were tested as Fenton-like homogeneous catalysts, analyzing the conversion, kinetics, and mechanistic insights of the MO oxidative decomposition (Figure 2). Then, the Fe(III) complexes that exhibited the best catalytic performance were selected as templates to imprint catalytic sites on a polymeric matrix, following a molecular imprinting technique. Finally, the catalytic activity of the MIPs was assessed in comparison to that shown by the corresponding homogeneous Fe(III) catalysts, allowing us to evaluate the impact of the polymeric matrix.

## 2. Materials and Methods

### 2.1. Chemicals

All chemicals used throughout this work were purchased from commercial sources and used without further purification. Iron(III) chloride hexahydrate (FeCl_3_·6H_2_O, Sigma-Aldrich, St. Louis, MO, USA, ≥ 97%) was used as a metal source. Tris(2-pyridylmethyl)amine (TPA, Sigma-Aldrich, St. Louis, MO, USA, 98%), di-(2-picolyl)amine (BMPA, Sigma-Aldrich, St. Louis, MO, USA, 97%), 1,4,8,11-tetraazacyclotetradecane (CYCLAM, Sigma-Aldrich, St. Louis, MO, USA, 98%), nitrilotriacetic acid (NTA, Sigma-Aldrich, St. Louis, MO, USA, 99%), and 3,3′,3″-nitrilotripropionic acid (NTP, Aldrich, Darmstadt, Germany) were used as ligands. The reagents for the molecular imprinting were methacrylamide (98%, Merck, Darmstadt, Germany) as functional monomer, 1,3,5-triacryloylhexahydro-1,3,5-triazine (TAT, Merck, Darmstadt, Germany, 98%), N,N-methylenebisacrylamide (MBAA, Merck, Darmstadt, Germany, 99%), and ethylene glycol dimethacrylate (EDMA, Merck, Darmstadt, Germany, 98%) as crosslinking agents (Figure S1) and K_2_S_2_O_8_ (Merck, Darmstadt, Germany, 99%) as a radical initiator.

### 2.2. MIPs Preparation and Characterization

The Fe(III)-complex-imprinted polymers were prepared as follows. The Fe(III) salt and ligand (0.10 mmol each) were dissolved in 5 mL of H_2_O. Then, an aqueous solution of methacrylamide (5 mL, 0.55 mmol) was added with stirring. The crosslinking agent (1 mmol) dissolved in 2 mL of solvent (ethanol for TAT or EDMA; water for MBAA) was incorporated with stirring. The O_2_ was displaced by bubbling with N_2_ for 10 min. The radical polymerization was initiated by adding 0.4 mmol of K_2_S_2_O_8_, then gently heating the solution on a plate at 60 °C with magnetic stirring for 24 h (heating plate, IKA, Staufen, Germany). The MIPs were filtered off, washed with 5 mL of CH_3_CN and 5 mL of water, and dried in an air oven at 85 °C for 30 min (SELECTA air oven, Laboquimia, Lardero, Spain). Non-imprinted polymers (NIPs) were also prepared following the same strategy but without the Fe(III) complex.

The polymers were characterized by infrared spectroscopy, preparing the samples as 1% KBr pellets and measuring the spectra with a Shimadzu IR Prestige 21 Fourier-transform infrared spectrophotometer (Shimadzu, Kyoto, Japan). Additionally, the Fe(III) content per gram of MIP was determined by flame atomic absorption spectrometry, using a Thermo iCE 3500 spectrometer (Thermo Fisher Scientific, Waltham, MA USA) operated at 248.33 nm. The flame composition was acetylene (Linde, Montevideo, Uruguay, 99.99%, 2.5 L min^−1^)–air (10.0 L min^−1^). Samples were previously subjected to a microwave-assisted acid digestion using a CEM Mars 6 microwave instrument (CEM, Matthews, NC USA). For this task, 50 mg of dried and ground sample was accurately weighted into each vessel and 10.0 mL of 3.5 mol L^−1^ HNO_3_ was added. The program consisted of a 15 min ramp time until 200 °C, holding for 20 min, and then cooling to room temperature.

### 2.3. Kinetic and Mechanistic Characterization in Solution

The kinetic analysis of the MO oxidative decomposition was performed as follows. A 10 mL aqueous solution containing the following total initial concentrations was prepared: MO (6 × 10^−5^ mol L^−1^), Fe^3+^ (1.9 × 10^−4^ mol L^−1^), and the corresponding ligand (1.9 × 10^−4^ mol L^−1^). The reaction was started by adding H_2_O_2_ (2.93 × 10^−3^ mol L^−1^), then the UV-vis spectra were monitored over time using a Thermo Scientific Evolution 60S UV-Visible spectrophotometer (Thermo Fisher Scientific, Waltham, MA USA). The catalytic runs were carried out for 180 min, following the protocol reported by Carvalho et al. [26]. That time interval was enough to differentiate the catalytic efficiency of our catalysts. During the experiments, the solution was stirred under isothermal conditions (20.0 ± 0.5 °C) using a Brookfield TC 2000 circulating bath (AMETEK Brookfield, Middleborough, MA, USA).

The conversion (degradation) of MO was determined by employing the following equation:

(1) $$\%\;\mathrm{conversion}=100\;\left[1-\left(\frac{A_{\max\mathrm{final}}}{A_{\max\mathrm{initial}}}\right)\right]$$

where $A_{\max\mathrm{initial}}$ and $A_{\max\mathrm{final}}$ are the MO maximum absorbance values at the beginning and the end of the experiment, respectively. It was verified in each case that no other species, including the Fe(III) complexes, significantly absorb light at this wavelength.

The kinetic parameters and mechanistic insights were obtained by fitting the spectroscopic data to three kinetic models, namely the first-order model [41], second-order model [41], and the one proposed by Carvalho et al. [26]. The corresponding equations are:

(2) $$\mathrm{First}-\mathrm{order}\;\mathrm{model}\text{:}\;C_{t}=C_{0}\;e^{-\left(k\right)t}$$

(3) $$\mathrm{Second}-\mathrm{order}\;\mathrm{model}\text{:}\;\frac{1}{C_{t}}-\frac{1}{C_{0}}=\left(k\right)t$$

(4) $$\mathrm{Carvalho}\;\mathrm{et}\;\mathrm{al}.\;\mathrm{model}\text{:}\;C_{t}=ae^{-(k_{1})t}+be^{-(k_{2})t}$$

where k, $k_{1}$, and $k_{2}$ are reaction rate constants; $C_{0}$ and $C_{t}$ are MO concentrations at reaction times 0 and *t*, respectively; and *a* and *b* are weight parameters. Equations (2) and (3) assume only one determining step, while the model proposed by Carvalho et al. [26] is based on the assumption that the following two first-order steps occur simultaneously:

MO → oxidized products (MO oxidized by L-Fe(III)-OOH; see Figure 1)  *k*_1_

MO → oxidized products (MO oxidized by OH^●^, HO_2_^●^, etc.; see Figure 1)  *k*_2_

In addition, the concentration of the intermediate L-Fe(III)-OOH (Figure 1) was monitored by registering the absorption shoulder around 320 nm, fitting the experimental data with the following equation proposed by Carvalho et al. [26]:

(5) $$C_{t}=c(1-e^{-(k_{3})t})+de^{-\left(k_{4}\right)t}$$

where *c* and *d* are weight parameters. This kinetic model assumes that the intermediate L-Fe(III)-OOH is formed and then reacts following two first-order successive steps (see Figure 1):

L-Fe(III)-OH + H_2_O_2_ → L-Fe(III)-OOH + H_2_O     *k*_3_

L-Fe(III)-OOH → products        *k*_4_

The oxidative catalytic behavior of the polymers was assessed by monitoring the UV-vis absorption spectra of a solution of MO and H_2_O_2_ in contact with the MIPs. In a typical experiment, the MIP (40 mg) was suspended in a 10 mL aqueous solution of MO (initial concentration = 6 × 10^−5^ mol L^−1^). The system was stirred magnetically and the temperature was adjusted at 20.0 ± 0.5 °C using a Brookfield TC 2000 circulating bath. The reaction was started by adding H_2_O_2_ (initial concentration = 2.93 × 10^−3^ mol L^−1^). The UV-vis spectra of the solution were monitored over time (180 min) using a Thermo Scientific Evolution 60S UV–visible spectrophotometer. To do so, the stirring was stopped and the supernatant was pressure-filtered into the quartz cuvette using a Merck Millipore filter (pore size = 0.45 µm; Merck, Darmstadt, Germany).

The catalytic activity of the MIPs may be due, at least partially, to the lixiviation of the Fe(III) ion/complex from the polymer into the MO solution. To evaluate this possibility, the Fe(III) concentration was determined in the supernatant by flame atomic absorption spectrometry as previously stated.

## 3. Results and Discussion

### 3.1. Fe(III) Complexes Catalytic Performance

As a first step in our approach (Figure 2), we proceeded to evaluate the catalytic behavior of five different Fe(III) complexes towards the oxidative degradation of MO, with the aim of selecting the best templates for the molecular imprinting stage. We explored five stable metal complexes bearing a range of different topologies, coordination numbers, geometries, and donor atoms, including Fe(III)-NTP, Fe(III)-TPA, Fe(III)-NTA, Fe(III)-CYCLAM, and Fe(III)-BMPA [42]. The latter was included since it was recently reported that this complex is particularly effective at catalyzing the oxidative discoloration of MO [26]. During the catalytic experiments, the iron(III) complexes were generated in situ in the aqueous solution from an equimolar mix of the metal ion and the ligands. This strategy was adopted instead of preparing and isolating the solid coordination compounds prior to its use, as it is usually done, to save time and costs.

**Figure 3:**
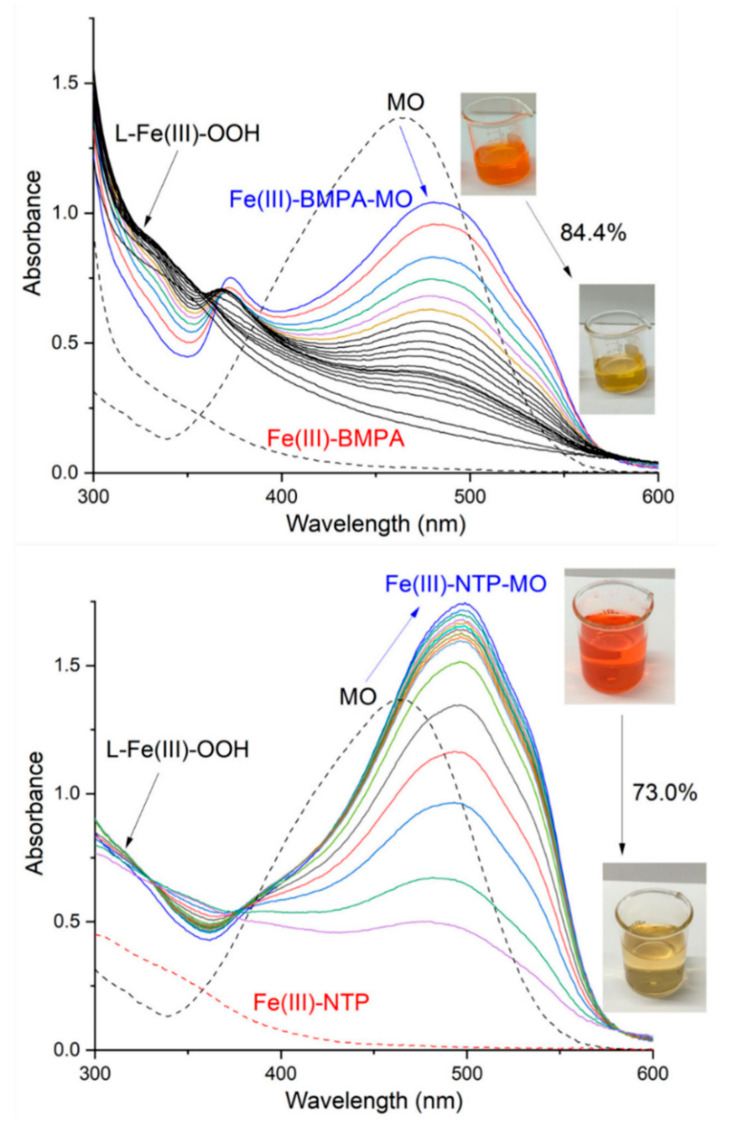
Time evolution of the UV-vis spectral profile for Fe(III)-BMPA (up) and Fe(III)-NTP (down) systems. Conditions: *T* = 20.0 °C; 3 h; [MO] = 6 × 10^−5^ mol L^−1^, [Fe] = [ligand] = 1.9 × 10^−4^ mol L^−1^, [H_2_O_2_] = 2.93 × 10^−3^ mol L^−1^. The MO conversion (%) is also included.

Under the conditions employed in the experiments (*T* = 20.0 °C; 3 h; [MO] = 6 × 10^−5^ mol L^−1^, [Fe] = [ligand] = 1.9 × 10^−4^ mol L^−1^, [H_2_O_2_] = 2.93 × 10^−3^ mol L^−1^), the iron(III) complexes of BMPA and NTP were catalytically more active, partially discoloring the MO (see Figure S2). To further characterize the catalytic systems, the UV-vis spectral profile was monitored during the experiments (Figure 3 and Figure S3). The results showed that in all cases, the MO absorption band was shifted and its maximum absorbance changed when it interacted with the iron(III) complex. This suggests that a pH change (MO is a pH indicator) or the formation of a ternary Fe(III)–ligand–MO complex occurred [26,43]. This absorption band has been ascribed to the azo bond and its decay is associated with the destruction of the MO chromophore [25]. Indeed, it has been proposed that the Fenton oxidation of methyl orange occurs via the rupture of the -N=N- group, followed by the hydroxylation of the aromatic rings. Some degradation products have been identified, including benzene, phenol, hydroquinone, 1,4-benzoquinone, pyrocatechol, nitrocatechol, 1,3,5-trihydroxynitrobenzene, p-nitrophenol, acetic acid, and CO_2_ [44,45]. As expected from the results in Figure S2, the absorbance of the azo band decreased significantly for Fe(III)-BMPA and Fe(III)-NTP (Figure 3), giving rise to high oxidative conversions (84.4 and 73.0% MO degradation, respectively). This catalytic behavior was due to the metal complexes, since the degradation fell to 1.2% in the absence of iron and ligands (Figure S4).

For the other complexes (Figure S3), the changes in the spectral profiles were much less noticeable, exhibiting lower MO degradation in 3 h for Fe(III)-TPA (28.0%) and Fe(III)-NTA (17.4%). The Fe(III)-CYCLAM complex, however, deserves special mention. The UV-vis absorption spectrum (Figure S5) showed the formation of at least one ternary species, Fe(III)-CYCLAM-MO, whose spectral profile changed with time. The absorbance of the band recorded at 376 nm decreased, while that observed at 464 nm increased, giving rise to isosbestic points at 336, 367, 388, and 469 nm. Strikingly, this phenomenon occurred even in the absence of H_2_O_2_ (Figure S5) and was accompanied by a decrease in pH from 5.62 to 4.87. This evidence suggests that the ternary species Fe(III)-CYCLAM-MO might not be highly stable and could undergo aquation over time, releasing MO. In fact, a similar behavior showing spectral profiles with various isosbestic points has been reported for *trans*-chloro CYCLAM metal complexes, being ascribed to aquation processes [46,47]. In any case, our spectroscopic evidence showed no substantial MO oxidative degradation during the catalytic experiments with Fe(III)-CYCLAM.

In conclusion, the iron complexes that exhibited the best catalytic performance were Fe(III)-BMPA and Fe(III)-NTP; therefore, they were selected as templates for the molecular imprinting stage (Figure 2). Interestingly, in the absence of ligands, the Fe^3+^ ion showed even better catalytic activity, with 94.7% MO degradation (Figure S3). In this sense, the metal coordination reduced the catalytic performance of the iron center. Of all the ligands tested, BMPA and NTP retained most of the catalytic activity of the metal cation, while providing coordinative bonds to keep the iron inside the MIPs [36,48,49]. It is feasible that during the molecular imprinting phase, methacrylamide and the crosslinkers establish hydrogen bonds with the N and O atoms of the ligands, along with other non-covalent interactions such as dispersion forces. After the polymerization, the Fe(III) complexes become trapped in the polymeric matrix that is formed around them, ending up in suitably shaped cavities.

### 3.2. Kinetic Parameters and Mechanistic Insights

**Figure 4:**
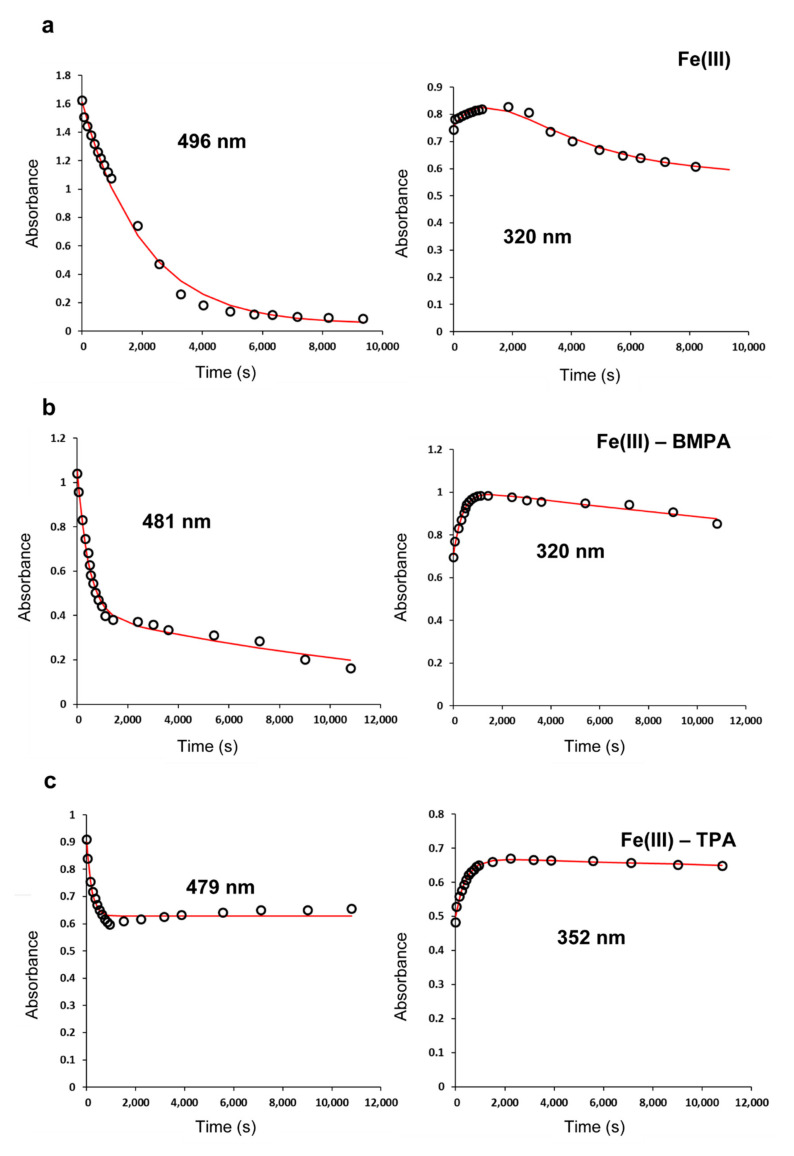

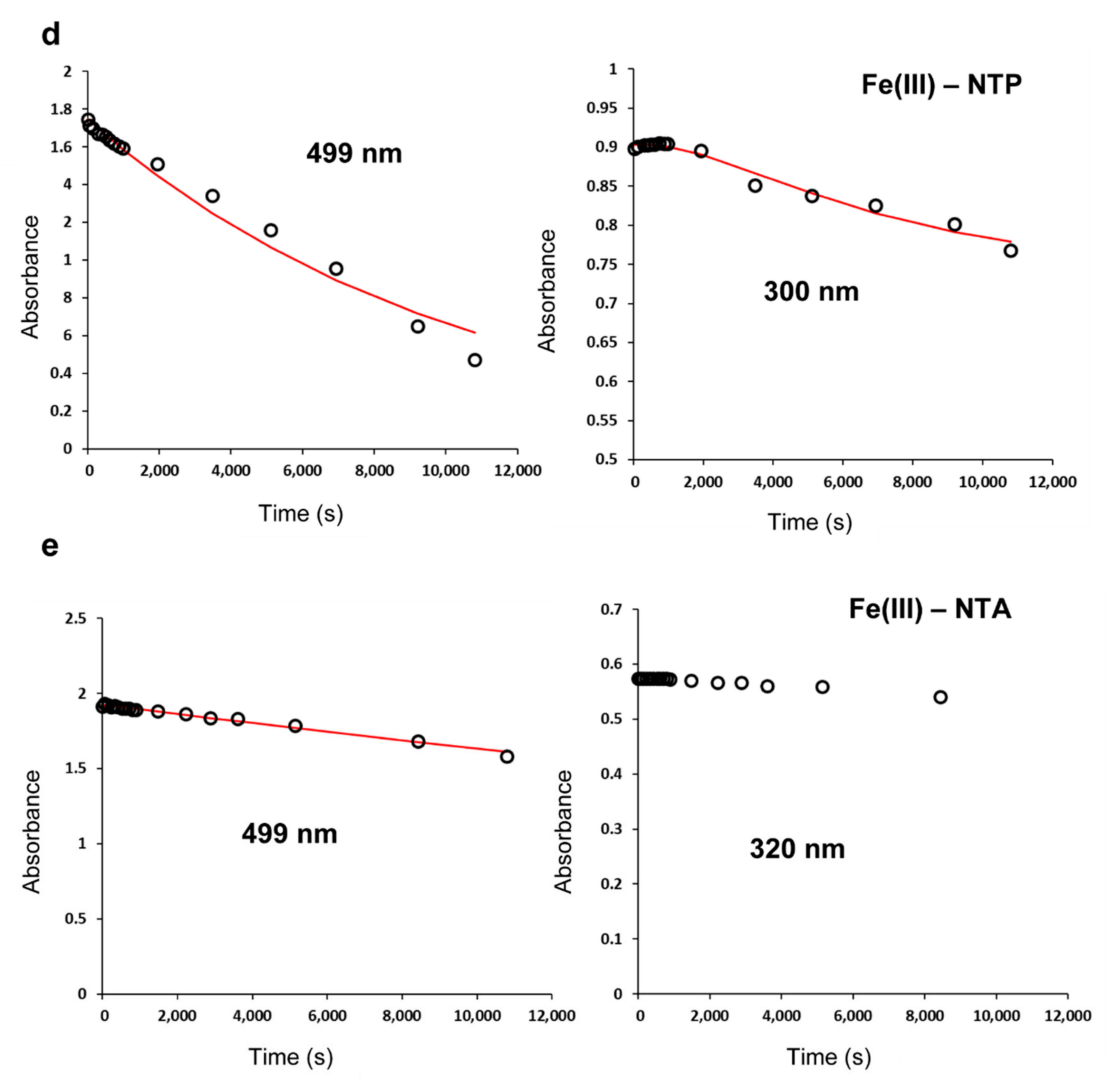
Time evolution of the absorbance for the MO degradation catalyzed by Fe(III) (**a**), Fe(III)-BMPA (**b**), Fe(III)-TPA (**c**), Fe(III)-NTP (**d**) and Fe(III)-NTA (**e**). The specific wavelength of measurement is also indicated. The optimal kinetic model is represented in each case as a red line. Conditions: *T* = 20.0 °C; 3 h; [MO] = 6 × 10^−5^ mol L^−1^, [Fe] = [ligand] = 1.9 × 10^−4^ mol L^−1^, [H_2_O_2_] = 2.93 × 10^−3^ mol L^−1^.

In order to deepen in the characterization of the Fe(III) homogeneous catalysts, we evaluated the kinetic parameters and mechanistic details of the MO oxidative degradation. The plots of MO maximum absorbance versus time (Figure 4) were fitted to three kinetic models, namely the first-order model, second-order model, and the one proposed by Carvalho et al. [26] (see the experimental section). The latter assumes two first-order steps occurring simultaneously, involving the degradation of MO through (i) oxidation by L-Fe(III)-OOH (*k*_1_, Figure 1) and (ii) oxidation by reactive oxygen species (ROS): OH^●^, HO_2_^●^, etc. (*k*_2_, Figure 1). Additionally, the intermediate L-Fe(III)-OOH (Figure 1) was monitored by following the absorption shoulder around 320 nm (Figure 3 and Figure S6), fitting its time evolution (plots in Figure 4) to an equation also proposed by Carvalho et al. [26]. This model assumes that this intermediate is formed (*k*_3_) and then consumed (*k*_4_) following two first-order successive steps (see Figure 1). The optimum model was selected in each case by determining the goodness of fit (*R*^2^). When *R*^2^ values were similar, the simplest model was chosen (Table S1). The results are listed in Table 1 and Figure 4.

In the absence of ligands, the Fe^3+^ ion catalyzed the oxidative degradation of MO via a first-order step with *k* = 0.027 min^−1^ (Table 1). It is feasible that the reaction occurred through the path involving the species Fe(III)-OOH (Figure 1), since its absorption band at 320 nm showed an increment and then a decrease in its absorbance (Figure 4a). Indeed, the initial pH was around 3.9, revealing a partial hydrolysis of the iron center, giving rise to Fe(III)-OH species [42] that are precursors of the intermediate Fe(III)-OOH. According to the best-fitted kinetic model, this intermediate was formed more rapidly than it was consumed (*k*_3_ > *k*_4_), having a maximum concentration value at around 30 min. No evidence of a second step related to reactive oxygen species was found in this case, since only one step associated with the Fe(III)-OOH intermediate could be fitted. As a matter of fact, it is known that Fe(III) ion generates ROS very slowly [33].

When one of the ligands was present in the solution, the scenario changed. Depending on the nature of the ligand, the MO degradation in 3 h was modulated between 17% and 84%. According to the kinetic modeling, the Fe(III)-BMPA complex catalyzed the reaction through two parallel first-order processes (*k*_1_ = 0.13 min^−1^; *k*_2_ = 0.0040 min^−1^), in line with the findings of Carvalho (*k*_1_ = 0.154 min^−1^; *k*_2_ = 0.012 min^−1^) [26]. The first process was related to the path involving the intermediate Fe-OOH, since the plot at 320 nm in Figure 4b shows the initial increase in Fe-OOH absorbance. Its formation (*k*_3_ = 0.16 min^−1^) occurred at a higher rate than in the absence of BMPA (*k*_3_ = 0.047 min^−1^), concentrating the intermediate and initially accelerating the MO degradation; however, this ligand also stabilized the intermediate Fe-OOH, making it somewhat less reactive (*k*_4_ = 0.0012 min^−1^) than that of Fe(III) (*k*_4_ = 0.023 min^−1^). Then, when the formation of Fe-OOH had slowed down, it began to decay and a second MO degradation route, attributed to the oxidation by hydroxyl radical [26], started to dominate (*k*_2_ = 0.0040 min^−1^). TPA ligand behaved similarly to BMPA, although this second step was significantly decelerated (*k*_1_ = 0.29 min^−1^; *k*_2_ = 1.8 × 10^−14^ min^−1^), suggesting that no ROS were generated. This was in agreement with the reported experimental evidence [50,51], which indicates that TPA iron complexes are among those that exhibit the highest catalytic activity during the dihydroxylation of organic compounds in acetonitrile. They act through a mechanism that is incompatible with the substantial generation of ROS, encompassing the sequential formation of Fe^III^ꟷOOH and *cis*-HOꟷFe^V^=O species [51,52].

On the other hand, the addition of NTP did not exert profound changes in the mechanistic aspects of the MO oxidative degradation. Indeed, the complex with this ligand behaved similarly to Fe^3+^, catalyzing the reaction via one dominant first-order step. Nevertheless, this path was much slower (*k*_NTP_ = 0.0058 min^−1^; Table 1) than for the iron(III) ion (*k* = 0.027 min^−1^; Table 1), giving an explanation for the lower degradation efficiency. The NTP ligand exhibited a Fe-OOH intermediate that was formed and consumed almost at the same speed (*k*_3_ = 0.019 min^−1^; *k*_4_ = 0.016 min^−1^), maintaining a steady level of MO consumption throughout the 3 h (Figure 4d). Conversely, NTA did not promote the formation of Fe-OOH species (Figure 4e), forcing the MO discoloration to go through the reaction with ROS (*k*_NTA_ = 0.00097 min^−1^, Figure 1).

Finally, it is worth noting that Fe(III)-CYCLAM did not lead to significant dye degradation. This possibly occurred because (i) the formation of the intermediate Fe(III)-OOH requires at least two labile positions in *cis* (see Figure 1) [50,51] and (ii) the complex with CYCLAM did not promote the formation of ROS such as OH^●^ and HO_2_^●^ to a large extent.

### 3.3. Fe(III)-Complex-Imprinted Polymers

As a second step in our experimental strategy (Figure 2), we set out to imprint polymeric matrices with the metal catalysts that exhibited the best performance, namely Fe-BMPA and Fe-NTP. The molecular imprinting was carried out in water with methacrlyamide as a polar functional monomer. Three crosslinking agents bearing different polarity and crosslinking ability levels were tried (EDMA, TAT, MBAA; Figure S1), so as to vary the chemical nature of the polymeric matrix. The results are listed in Table 2.

The six MIPs were obtained as white to brown-grey solids with masses ranging between 175 and 474 mg. All of them contained iron (1.26–11.13 mg/g), providing proof of the immobilization of the metal complexes inside the polymers. The chemical nature of the matrices was further analyzed by infrared spectroscopy (Table S2, Figures S7–S12), since it gives useful information on the presence of expected functional groups and the types of bonds existing in the MIP [53]. All of the polymers exhibited a broad and intense band between 3100 and 3700 cm^−1^, ascribed to the stretching of O-H and N-H bonds present in the methacrylamide moieties and the water molecules trapped in the solid structure. The broadening of this band is indicative of extensive hydrogen bonding, operative between these polar groups [54]. In all cases, the spectra showed an intense peak at around 1662–1732 cm^−1^, brought about by the stretching of the carbonyl C=O groups coming from the polymerization reagents (Figure S1). For the polymers containing EDMA, the C=O band appeared in conjunction with the C-O stretching (1157 and 1253–1257 cm^−1^), a feature that has been reported as an indication of poly(EDMA) as a cross-linker [53]. As expected, the C=C stretching band that was visible between 1608 and 1639 cm^−1^ for the polymerization reagents was absent in the MIP spectra.

### 3.4. MIPs as Fenton-like Heterogeneous Catalysts

**Figure 5:**
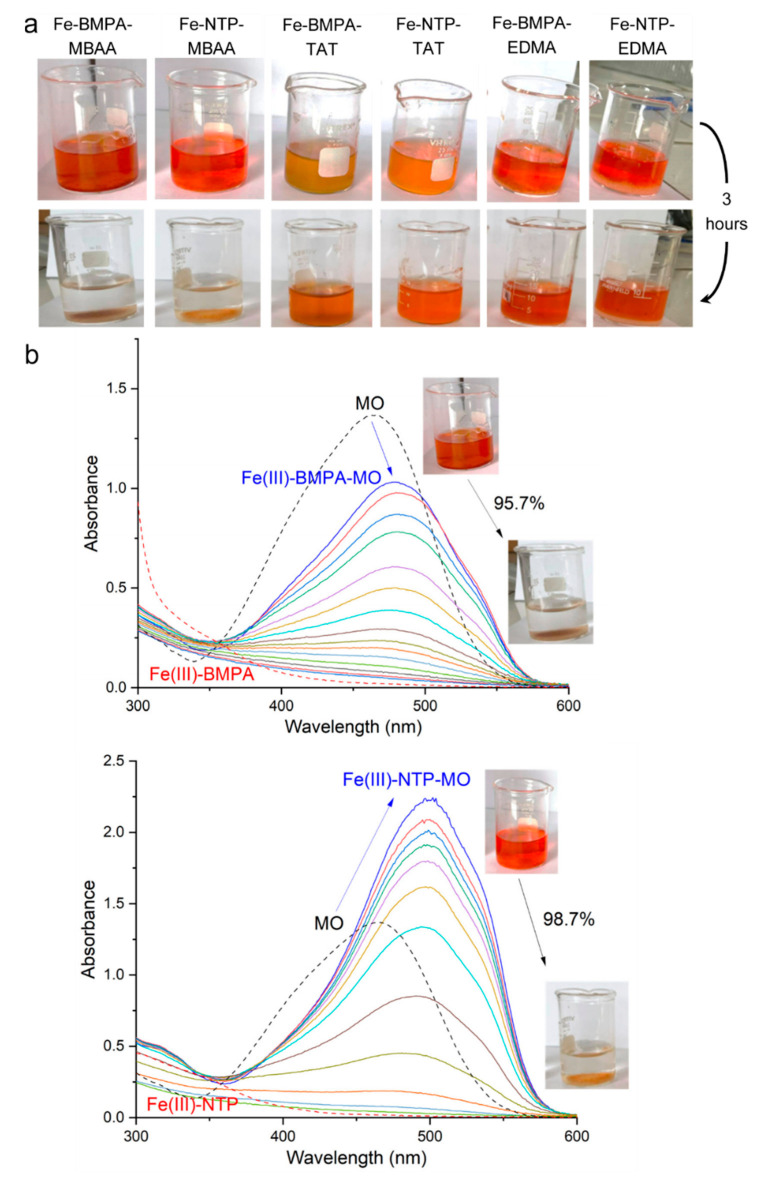
Catalytic performance of the Fe(III)-complex-imprinted polymers towards the oxidative degradation of MO in the presence of H_2_O_2_. (**a**) Change in the color of the solutions during the experiments. (**b**) Time evolution of the supernatant UV-vis spectral profile for Fe(III)-BMPA-MBAA and Fe(III)-NTP-MBAA systems. Conditions: *T* = 20.0 °C; 3 h; [MO] = 6 × 10^−5^ mol L^−1^, MIP mass = 40 mg, [H_2_O_2_] = 2.9 × 10^−3^ mol L^−1^. The MO conversion (%) is also included.

As the final step in our study, the imprinted polymers were evaluated as Fenton-like heterogeneous catalysts towards the MO oxidative degradation. During the experiments, the MIPs were suspended in identical dye–H_2_O_2_ solutions with magnetic stirring at 20.0 °C. The overall results after 3 h are depicted in Figure 5a for all systems and show an almost complete discoloration of the MO only for the MBAA-containing polymers.

Table 2 lists the quantitative measure of the degradation efficiency for the six MIPs. In line with results in Figure 5a, the polymers crosslinked with MBAA displayed the highest % conversion of 95.7% and 98.7%. The rest of the catalysts, however, exhibited much less efficiency, with MO degradation below 42%. In this regard, the nature of the crosslinker agent did exert an impact on the heterogeneous catalysts. A feasible explanation could be that MBAA rendered the polymers as more polar and flexible. A higher hydrophilic character possibly facilitated the entrance of the dye into the catalytic cavities inside the polymer matrix. Indeed, MBAA is considered a hydrophilic crosslinking agent that can be used to enhance the water compatibility and flexibility of the MIPs [38,55]. EDMA MIPs, with lower hydrophilicity, displayed less catalytic activity (28.4% and 41.8%). On the other hand, TAT polymers displayed the lowest catalytic activity of all the MIPs tested (23.5% and 7.6%), probably due to a high level of crosslinking that led to a smaller pore size. As a matter of fact, TAT is considered an hexafunctional crosslinker, because it has three reactive double bonds rather than two, as in the case of MBAA or EDMA, leading to higher crosslinking ability. This is why, when MBAA and TAT are comparatively analyzed, the degree of crosslinking is kept constant by reducing the TAT concentration on an equivalent double bond basis [56]. In this context, the degradation efficiency of the polymers seemed not to be directly related to their iron content (see values in Table 2), but instead associated with their hydrophilicity, flexibility, and crosslinking level. In spite of this, it is worth pointing out that for EDMA or TAT polymers (i.e., keeping the polymeric matrix constant), the catalytic activity was higher for those MIPs that had more iron; therefore, it is feasible that for these matrices, the catalysis happened mostly inside the solid phase.

Figure 5b shows how the UV-vis absorption spectra of the supernatants changed during the catalytic experiments with the MBAA MIPs. The MO absorption band decayed steadily, leading to the complete discoloring of the solution. This catalytic ability was due to the metal complex, since no dye decomposition was registered in the absence of the iron(III) complex (see the results for the non-imprinted polymer in Figure S13). This happened for all MIPs tested, for which the corresponding non-imprinted polymers (NIPs) displayed catalytic performances below 6% (see all degradation percentages in Table S3). Another interesting aspect to discuss is the fact that both polymeric catalysts displayed efficiencies above their homogeneous counterparts (compare Table 1 and Table 2). This finding pointed to a positive influence of the polymeric matrix on the catalytic activity. It could be argued that the polymeric matrix could adsorb or absorb the MO, extracting it from the solution and causing an additional increment in the calculated % conversion; however, this seemed not to be the case, since the UV-vis absorption spectra did not change in the absence of H_2_O_2_ (Figure S14).

Lastly, to go deeper into the mechanism through which the MIPs catalyze the MO degradation, we determined the iron content in the supernatant after the catalytic runs. The polymers released to the solution between 0.1% and 27% of their iron content (Table 2). The iron leaching was variable and there was no evident correlation with the chemical nature of the imprinted polymers (crosslinker, iron complex, iron content); therefore, a part of the catalysis might occur outside the polymeric matrix. Nevertheless, this “homogeneous contribution” to the total catalysis seemed not to be significant, since the degradation efficiency was not globally correlated to the concentration of Fe leached. In fact, even though the polymer EDMA-Fe-BMPA displayed the highest iron lixiviation during the catalytic runs, it promoted the degradation of only 28.4% of the initial MO. For both MBAA MIPs, which also showed high levels of iron release, the homogeneous catalysis did not account for the very high conversions, since the iron concentration in the supernatant was below 13% of that employed for the catalytic Fe(III) complexes ([Fe] = [ligand] = 1.9 × 10^−4^ mol L^−1^; Figure 3). In this sense, it is likely that both MBAA MIPs catalyzed the MO degradation mainly inside the polymeric phase, accumulating the dye near the catalytic sites. This led to the enhanced catalytic efficiency with respect to the corresponding Fe(III) complexes in the solution.

## 4. Conclusions

In this work, we aimed to imprint polymers with iron(III) complexes to produce efficient Fenton-like heterogeneous catalysts. Among the selected systems, the best-performing iron complexes were Fe(III)-BMPA and Fe(III)-NTP, which displayed above 70% MO degradation within 3 h. Fe(III)-BMPA caused the oxidative degradation through two first-order parallel stages related to the formation of BMPA-Fe-OOH species and the generation of reactive oxygen species. Regarding the complex with NTP, only the former stage was detected. The imprinted polymers prepared from both Fe(III) complexes showed catalytic profiles that were highly dependent on the crosslinking agent employed. MBAA was the crosslinker that rendered polymers with optimal oxidative performance (>95% conversions), possibly because it led to the formation of more hydrophilic and flexible polymers that favored the entrance and accumulation of the dye into the catalytic cavities. Conversely, EDMA- and TAT-containing MIPs showed lower catalytic performance. For these, the evidence suggested that a main part of the catalytic process occurred inside the polymeric matrix, with the dye entrance to the catalytic sites probably being hampered by low polarity or high crosslinking.

Both MBAA MIPs showed excellent catalytic performance. These samples constitute the first examples of highly promising materials that can be used in the quest for efficient and low-cost Fenton-like ion-imprinted catalysts, which could be coupled to dye wastewater treatment systems working under synchronous injection of H_2_O_2_; however, further studies are needed to achieve this goal, focusing on the cost, robustness, and recyclability of the MIPs. We are currently undertaking some studies to explore the impacts of parameters such as pH, cycles of use, and the nature of the target dye on the MIPs catalytic performance. The results will be published in due course.

## Figures and Tables

**Table 1:** Adjusted kinetic parameters (±SD) for the MO oxidative degradation by H_2_O_2_.

Catalyst	Initial pH	Optimum Kinetic Model ^a^							Conversion (%) (3 h)
		MO Degradation				Intermediate L-Fe(III)-OOH			
		*R* ^2^	First-Order	Carvalho et al.		*R* ^2^	Carvalho et al.		
			$A=A_{0}e^{-kt}$	$A=ae^{-k_{1}t}+be^{-k_{2}t}$			$A=a\left(1-e^{-k_{3}t}\right)+be^{-k_{4}t}$		
			*k* (min^−1^)	*k*_1_ (min^−1^)	*k*_2_ (min^−1^)		*k*_3_ (min^−1^)	*k*_4_ (min^−1^)	
Fe(III)	3.9	0.99	0.027 ± 0.001	---	---	0.99	0.047 ± 0.003	0.023 ± 0.001	94.7
Fe–BMPA	4.6	0.99	---	0.13 ± 0.01	0.0040 ± 0.0007	0.98	0.16 ± 0.01	0.0012 ± 0.0001	84.4
Fe–TPA	3.8	0.96	---	0.29 ± 0.04	1.8 × 10^−14 c^	0.99	0.14 ± 0.01	0.00025 ± 0.00009	28.0
Fe–NTP	3.4	0.98	0.0058 ± 0.0003	---	---	0.98	0.019 ± 0.005	0.016 ± 0.004	73.0
Fe–NTA	3.5	0.99	0.00097 ± 0.00005	---	---	--- ^b^	---	---	17.4

^a^ A = MO maximum absorbance. ^b^ The absorbance variation at 320 nm is negligible. ^c^ Value with high uncertainty (σ = 0.0002).

**Table 2:** Mass, total iron content, total iron leaching, and MO degradation efficiency of the Fe(III)-complex-imprinted polymers.

Polymeric Matrix	Template	Mass (mg)	Fe Content (mg/g MIP) ^a^	Fe Released (mg/g MIP) ^a^	Fe Released (%)	MO Degradation (%) (3 h)
MIP-MBAA	Fe-BMPA	175	2.41 ± 0.01	0.31 ± 0.05	12.9	95.7
	Fe-NTP	255	1.33 ± 0.06	0.23 ± 0.02	17.3	98.7
MIP-EDMA	Fe-BMPA	474	1.26 ± 0.06	0.342 ± 0.001	0.1	28.4
	Fe-NTP	226	11.13 ± 0.03	0.18 ± 0.03	5.3	41.8
MIP-TAT	Fe-BMPA	253	9.42 ± 0.15	0.012 ± 0.001	27.1	23.5
	Fe-NTP	279	1.54 ± 0.03	0.082 ± 0.001	1.6	7.6

^a^ Iron contents expressed as mean values ± standard deviation, *n* = 3.

## Data Availability

The data presented in this study are available on request from the corresponding author.

## Supplementary Materials

The following are available online at https://www.mdpi.com/article/10.3390/polym13183127/s1: Figure S1: Crosslinking agents used in this work. Figure S2: Changes in the color of the solutions during the catalytic experiments with the Fe(III) complexes. Figure S3: Time evolution of the UV-vis spectral profiles during the catalytic experiments. Figure S4: Time evolution of the UV-vis spectral profiles during the catalytic experiments in the absence of iron and ligands. Figure S5: Time evolution of the UV-vis spectral profiles during the catalytic experiments for the systems containing Fe(III)-CYCLAM. Figure S6. Spectra deconvolution for Fe(III)-BMPA-MO and Fe(III)-NTP systems. Figure S7-S12: Infrared spectra of MIPs. Figure S13: Catalytic performance of the MBAA NIP towards the oxidative degradation of MO in the presence of H_2_O_2_. Figure S14: Catalytic performance of the Fe(III)-complex-imprinted polymers towards the oxidative degradation of MO in the absence of H_2_O_2_. Table S1: Fitting of the kinetic models. Table S2: Most important infrared bands of the Fe(III)-complex-imprinted polymers.

## References

[1] Uday U.S.P., Bandyopadhyay T.K., Bhunia B. (2016). Bioremediation and detoxification technology for treatment of dye(s) from textile efuent. Textile Wastewater Treatment.

[2] Yagub M.T., Sen T.K., Afroze S., Ang H.M. (2014). Dye and its removal from aqueous solution by adsorption: A review. Adv. Colloid Interface Sci..

[3] Allen S.J., McKay G., Porter J.F. (2004). Adsorption isotherm models for basic dye adsorption by peat in single and binary component systems. J. Colloid Interface Sci..

[4] Brindley L. (2009). New Solution for Dye Wastewater Pollution. Chemistry World. https://www.chemistryworld.com/news/new-solution-for-dye-wastewater-pollution/3002870.article.

[5] GEA Consultores Ambientales (2005). Gestión Ambiental en el Sector Secundario.

[6] Lalanne A., Carsen A., Lorenzo D., Perdomo A., Arriola M. (2005). Diagnóstico de Oportunidades de Implantación del Proyecto Piloto en Uruguay.

[7] Chen S., Zhang J., Zhang C., Yue Q., Li Y., Li C. (2010). Equilibrium and kinetic studies of methyl orange and methyl violet adsorption on activated carbon derived from Phragmites australis. Desalination.

[8] Javaid R., Qazi U.Y. (2019). Catalytic Oxidation Process for the Degradation of Synthetic Dyes: An Overview. Int. J. Environ. Res. Public Health.

[9] Katheresan V., Kansedo J., Lau S.Y. (2018). Efficiency of various recent wastewater dye removal methods: A review. J. Environ. Chem. Eng..

[10] Robinson T., McMullan G., Marchant R., Nigam P. (2001). Remediation of dyes in textile effluent: A critical review on current treatment technologies with a proposed alternative. Bioresour. Technol..

[11] Forgacs E., Cserháti T., Oros G. (2004). Removal of synthetic dyes from wastewaters: A review. Environ. Int..

[12] Nikfar S., Jaberidoost M., Wexler P. (2014). Dyes and Colorants. Encyclopedia of Toxicology.

[13] Ahmadpour A. (2015). Photocatalytic decolorization of methyl orange dye using nano-photocatalysts. Adv. Environ. Technol..

[14] Budavari S. (1996). The Merck Index. Encyclopedia of Chemicals, Drugs, and Biologicals; p. 1537. https://www.worldcat.org/title/merck-index-an-encyclopedia-of-chemicals-drugs-and-biologicals/oclc/34552962.

[15] Heukelekian H., Rand M.C. (1955). Biochemical Oxygen Demand of Pure Organic Compounds: A Report of the Research Committee, FSIWA. Sew. Ind. Wastes.

[16] Chung K.-T., Fulk G.E., Andrews A.W. (1978). The mutagenicity of methyl orange and metabolites produced by intestinal anaerobes. Mutat. Res./Genet. Toxicol..

[17] Singh O., Maji A., Singh U.P., Ghosh K. (2018). Water-Soluble Copper Complex Derived from Ligand TETATA Having NNN Donors: Studies on Rapid Degradation of Organic Dyes, Catecholase and Phenoxazinone Synthase Activities. ChemistrySelect.

[18] Ajmal A., Majeed I., Malik R.N., Idriss H., Nadeem M.A. (2014). Principles and mechanisms of photocatalytic dye degradation on TiO_2_ based photocatalysts: A comparative overview. RSC Adv..

[19] Gupta V.K., Kumar R., Nayak A., Saleh T.A., Barakat M.A. (2013). Adsorptive removal of dyes from aqueous solution onto carbon nanotubes: A review. Adv. Colloid Interface Sci..

[20] Ahmad A., Mohd-Setapar S.H., Chuong C.S., Khatoon A., Wani W.A., Kumar R., Rafatullah M. (2015). Recent advances in new generation dye removal technologies: Novel search for approaches to reprocess wastewater. RSC Adv..

[21] Moursy A.S., Abdel-Shafy H.I. (1983). Removal of hydrocarbons from Nile water. Environ. Int..

[22] Alebic-Juretic A., Cvitas T., Klasinc L. (1990). Heterogeneous polycyclic aromatic hydrocarbon degradation with ozone on silica gel carrier. Environ. Sci. Technol..

[23] Haritash A.K., Kaushik C.P. (2009). Biodegradation aspects of Polycyclic Aromatic Hydrocarbons (PAHs): A review. J. Hazard. Mater..

[24] Manariotis I.D., Karapanagioti H.K., Chrysikopoulos C.V. (2011). Degradation of PAHs by high frequency ultrasound. Water Res..

[25] Chahbane N., Popescu D.L., Mitchell A., Chanda A., Lenoir D., Ryabov A.D., Schramm K.W., Collins T.J. (2007). FeIII–TAML-catalyzed green oxidative degradation of the azo dye Orange II by H2O2 and organic peroxides: Products, toxicity, kinetics, and mechanisms. Green Chem..

[26] Carvalho S.S.F., Carvalho N.M.F. (2019). Degradation of organic dyes by water soluble iron(III) mononuclear complexes from bis-(2-pyridylmethyl)amine NNN-derivative ligands. Inorg. Chem. Commun..

[27] Feng Y., England J., Que L. (2011). Iron-Catalyzed Olefin Epoxidation and cis-Dihydroxylation by Tetraalkylcyclam Complexes: The Importance of cis-Labile Sites. ACS Catal..

[28] Oldenburg P.D., Mas-Ballesté R., Que L., Oyama S.T. (2008). Bio-Inspired Iron-Catalyzed Olefin Oxidations: Epoxidation Versus cis-Dihydroxylation. Mechanisms in Homogeneous and Heterogeneous Epoxidation Catalysis.

[29] Carvalho N.M.F., Horn A., Antunes O.A.C. (2006). Cyclohexane oxidation catalyzed by mononuclear iron(III) complexes. Appl. Catal. A Gen..

[30] Silva G.C., Carvalho N.M.F., Horn A., Lachter E.R., Antunes O.A.C. (2017). Oxidation of aromatic compounds by hydrogen peroxide catalyzed by mononuclear iron(III) complexes. J. Mol. Catal. A Chem..

[31] Xiao J., Wang C., Lyu S., Liu H., Jiang C., Lei Y. (2016). Enhancement of Fenton degradation by catechol in a wide initial pH range. Sep. Purif. Technol..

[32] Bai C., Xiao W., Feng D., Xian M., Guo D., Ge Z., Zhou Y. (2013). Efficient decolorization of Malachite Green in the Fenton reaction catalyzed by [Fe(III)-salen]Cl complex. Chem. Eng. J..

[33] Xu H.-Y., Wang Y., Shi T.-N., Zhao H., Tan Q., Zhao B.-C., He X.-L., Qi S.-Y. (2018). Heterogeneous Fenton-like discoloration of methyl orange using Fe3O4/MWCNTs as catalyst: Kinetics and Fenton-like mechanism. Front. Mater. Sci..

[34] Lan H., Wang A., Liu R., Liu H., Qu J. (2015). Heterogeneous photo-Fenton degradation of acid red B over Fe2O3 supported on activated carbon fiber. J. Hazard. Mater..

[35] Wang Y., Liu C.S., Li F.B., Liu C.P., Liang J.B. (2009). Photodegradation of polycyclic aromatic hydrocarbon pyrene by iron oxide in solid phase. J. Hazard. Mater..

[36] Shilpa E.R., Gayathri V. (2018). Polymer immobilized Fe(III) complex of 2-phenylbenzimidazole: An efficient catalyst for photodegradation of dyes under UV/Visible light irradiation. J. Saudi Chem. Soc..

[37] Huang D., Wang C., Song Y. (2013). Immobilized complexes of the salen Schiff’s base with metal as oxidation catalysts. Russ. J. Gen. Chem..

[38] Haupt K., Linares A.V., Bompart M., Bui B.T.S. (2012). Molecularly imprinted polymers. Molecular Imprinting.

[39] Sun W., Tan R., Zheng W., Yin D. (2013). Molecularly imprinted polymer containing Fe(III) catalysts for specific substrate recognition. Cuihua Xuebao/Chin. J. Catal..

[40] Chen Z., Huang S., Zhao M., Li S., Cao S., Piletsky S.A., Turner A.P.F. (2016). Molecularly Imprinted Polymers for Biomimetic Catalysts. Molecularly Imprinted Catalysts.

[41] Cornish-Bowden A. (2013). Fundamentals of Enzyme Kinetics.

[42] (2013). The IUPAC Stability Constants Database, SC-Database, 5.83.

[43] Theodoridis A., Maigut J., Puchta R., Kudrik E.V., Van Eldik R. (2008). Novel iron(III) porphyrazine complex. Complex speciation and reactions with NO and H2O2. Inorg. Chem..

[44] Arshadi M., Abdolmaleki M.K., Mousavinia F., Khalafi-Nezhad A., Firouzabadi H., Gil A. (2016). Degradation of methyl orange by heterogeneous Fenton-like oxidation on a nano-organometallic compound in the presence of multi-walled carbon nanotubes. Chem. Eng. Res. Des..

[45] Guivarch E., Trevin S., Lahitte C., Oturan M.A. (2003). Degradation of azo dyes in water by Electro-Fenton process. Environ. Chem. Lett..

[46] Campi E., Ferguson J., Tobe M.L. (1970). Mechanism and steric course of octahedral aquation. XIII. Kinetics and steric course of the acid and base hydrolysis of cis- and trans-dichloro(1,4,8,11-tetraazacyclotetradecane)chromium(III) cations. Inorg. Chem..

[47] Wagenknecht P.S., Hu C., Ferguson D., Nathan L.C., Hancock R.D., Whitehead J.R., Wright-Garcia K., Vagnini M.T. (2005). Effects of Steric Constraint on Chromium(III) Complexes of Tetraazamacrocycles, 2. Comparison of the Chemistry and Photobehavior of the trans-Dichloro- and trans-Dicyano-Complexes of Cyclam, 1,4-C2-Cyclam, and 1,11-C3-Cyclam. Inorg. Chem..

[48] Roushani M., Beygi T.M., Saedi Z. (2016). Synthesis and application of ion-imprinted polymer for extraction and pre-concentration of iron ions in environmental water and food samples. Spectrochim. Acta A.

[49] Mitreva M., Dakova I., Karadjova I. (2017). Iron(II) ion imprinted polymer for Fe(II)/Fe(III) speciation in wine. Microchem. J..

[50] Olivo G., Cussó O., Borrell M., Costas M. (2017). Oxidation of alkane and alkene moieties with biologically inspired nonheme iron catalysts and hydrogen peroxide: From free radicals to stereoselective transformations. JBIC J. Biol. Inorg. Chem..

[51] Chen K., Costas M., Kim J., Tipton A.K., Que L. (2002). Olefin cis-dihydroxylation versus epoxidation by non-heme iron catalysts: Two faces of an FeIII-OOH coin. J. Am. Chem. Soc..

[52] McDonald A.R., Que L. (2011). Elusive iron(V) species identified. Nat. Chem..

[53] Yusof N.A., Rahman S.K.A.B., Hussein M.Z., Ibrahim N.A. (2013). Preparation and characterization of molecularly imprinted polymer as SPE sorbent for melamine isolation. Polymers.

[54] Noorhidayah I., Mohd Noor A., Azalina Mohamed N., Islam A.K.M.S. (2015). Computational Modeling and Synthesis of Molecular Imprinted Polymer for Recognition of Nitrate Ion. Malays. J. Anal. Sci..

[55] Yoshimi Y., Ishii N. (2015). Improved gate effect enantioselectivity of phenylalanine-imprinted polymers in water by blending crosslinkers. Anal. Chim. Acta.

[56] Patras G., Qiao G.G., Solomon D.H. (2000). Characterization of the pore structure of aqueous three-dimensional polyacrylamide gels with a novel cross-linker. Electrophoresis.

