# Supplementary material for: Fe(III)-Complex-Imprinted Polymers for the Green Oxidative Degradation of the Methyl Orange Dye Pollutant
Source: Polymers (Basel). 2021 Sep 16;13(18):3127. doi: 10.3390/polym13183127 (PMC8468195; doi:10.3390/polym13183127)
Supplement: Supplementary file 1 [file polymers-13-03127-s001.zip › polymers-1388071-supplementary.pdf]

Fe(III) complex imprinted polymers for the green oxidative degradation of the methyl orange dye pollutant

Paulina Haller, Ignacio Machado, Julia Torres, Agustina Vila and Nicolás Veiga*

Supplementary information

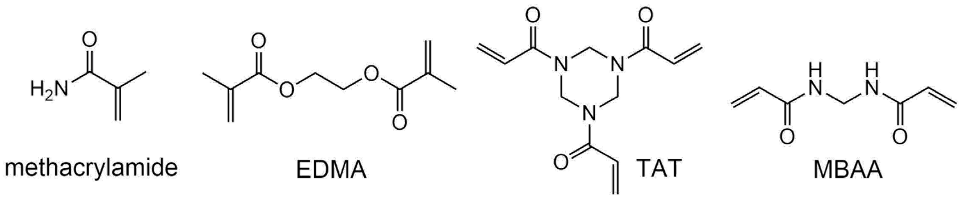

Figure S1. Crosslinking agents used in this work.

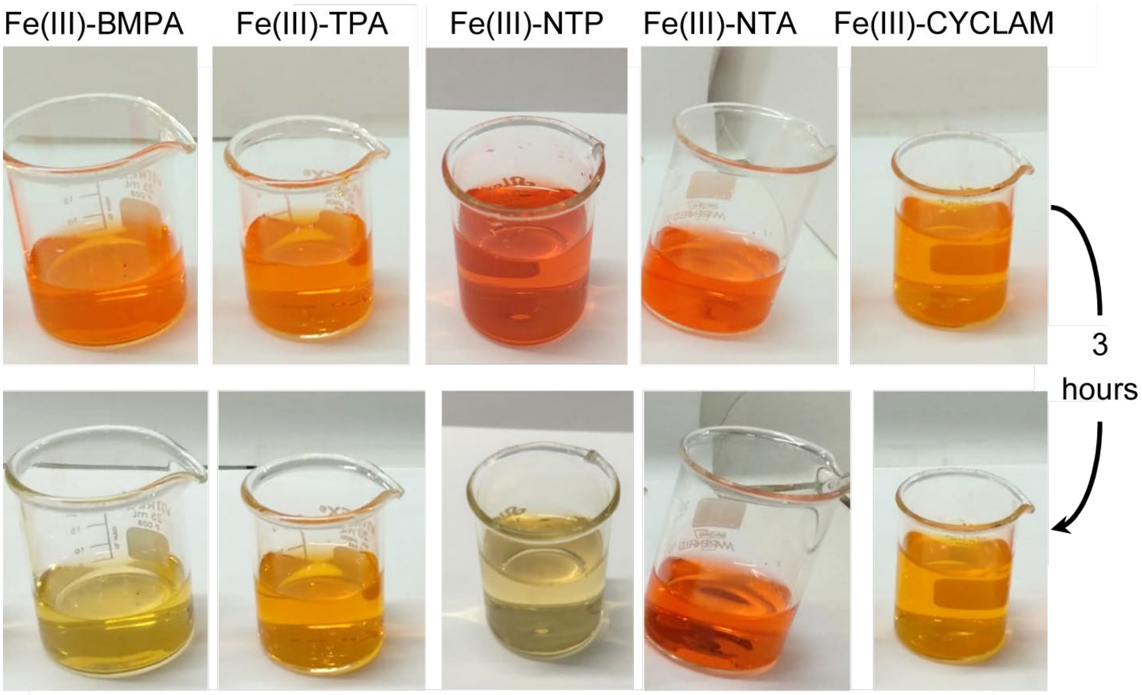

Figure S2. Change in the colour of the solutions during the catalytic experiments with the Fe(III) complexes. Conditions: *T* = 20.0 °C; 3 hours; [MO] = 6 x 10^{-5} mol L^{-1}, [Fe] = [ligand] = 1.9 x 10^{-4} mol L^{-1}, [H_2O_2] = 2.93 x 10^{-3} mol L^{-1}.

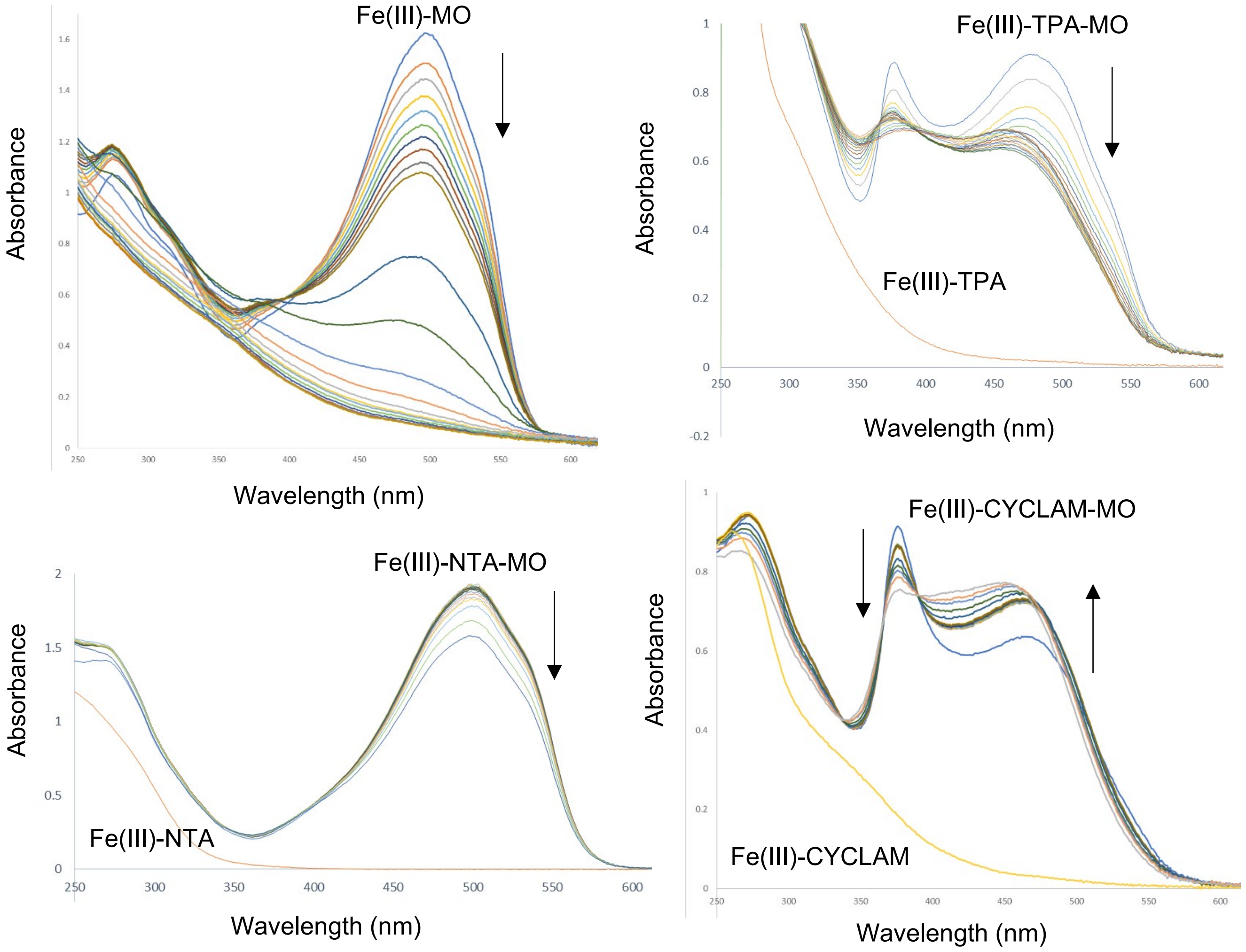

Figure S3. Time evolution of the UV-vis spectral profile during the catalytic experiments for the systems containing Fe^{3+}, Fe(III)-TPA, Fe(III)-NTA or Fe(III)-CYCLAM. Conditions: T = 20,0 °C; 3 hours; [MO] = 6 x 10^{-5} mol L^{-1}, [Fe] = [ligand] = 1.9 x 10^{-4} mol L^{-1}, [H_2O_2] = 2.93 x 10^{-3} mol L^{-1}.

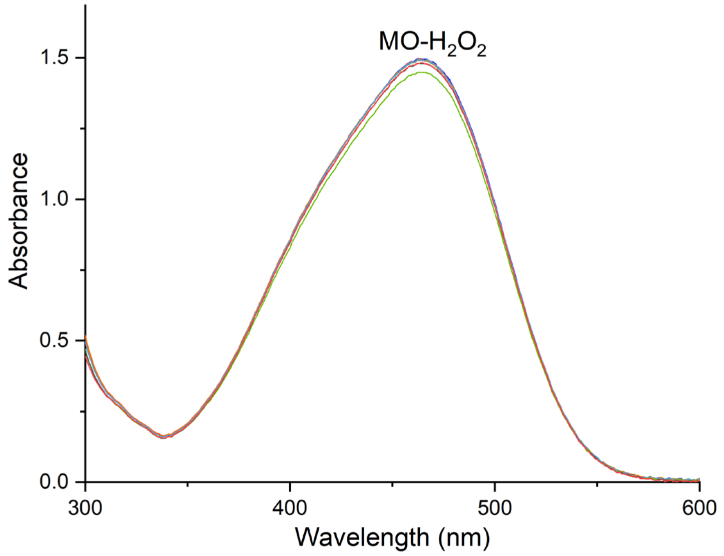

Figure S4. Time evolution of the UV-vis spectral profile during the catalytic experiments in the absence of iron and ligands. Conditions: T = 20,0 °C; 3 hours; [MO] = 6 x 10^{-5} mol L^{-1}, [H_2O_2] = 2.93 x 10^{-3} mol L^{-1}.

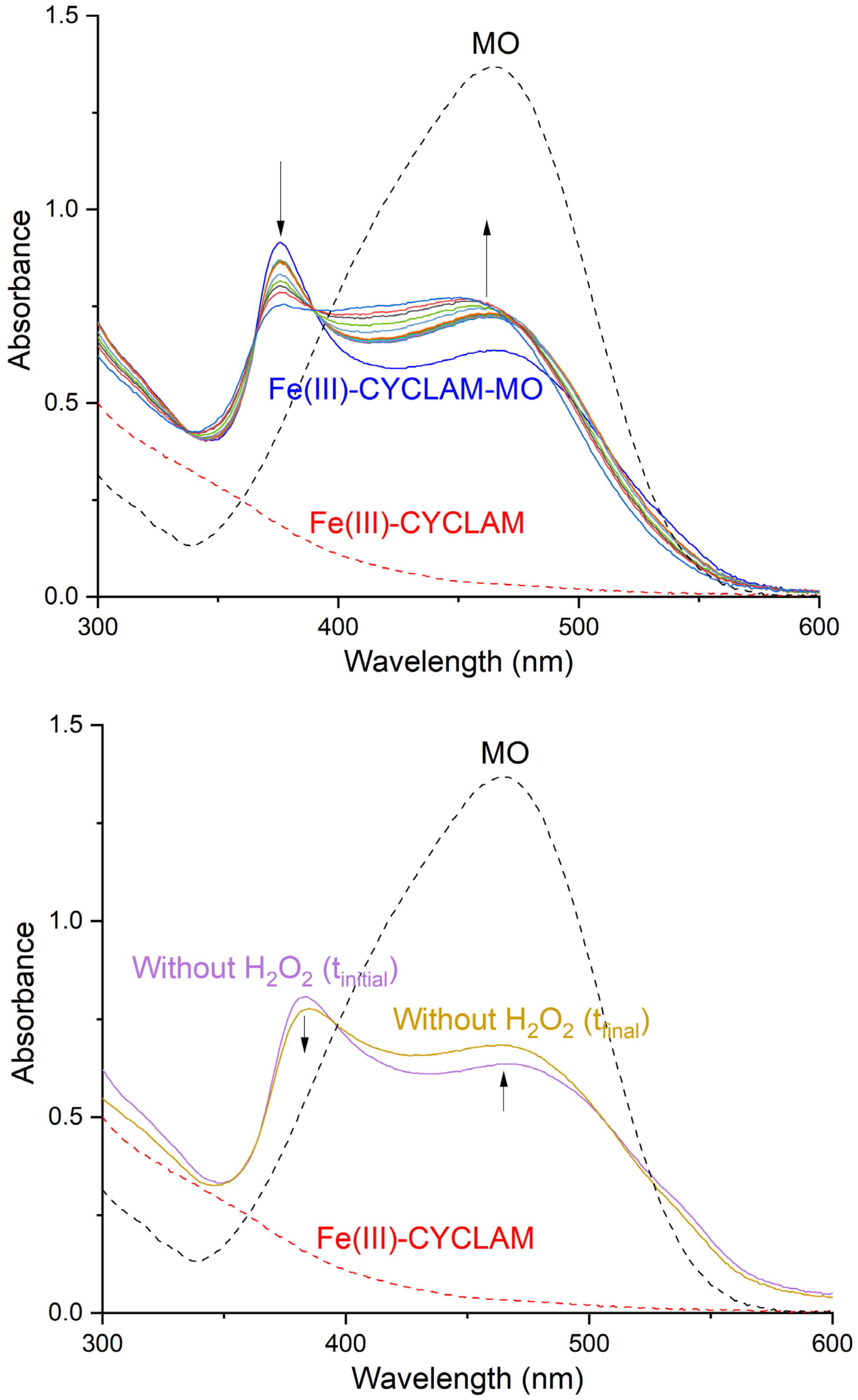

Figure S5. Up: Time evolution of the UV-vis spectral profile during the catalytic experiments for the systems containing Fe(**III**)-CYCLAM. Conditions: T = 20,0 °C; 3 hours; [MO] = 6 x 10^{-5} mol L^{-1}, [Fe] = [CYCLAM] = 1.9 x 10^{-4} mol L^{-1}, [H_2O_2] = 2.93 x 10^{-3} mol L^{-1}. Down: Initial and final spectra under the same conditions but in the absence of H_2O_2.

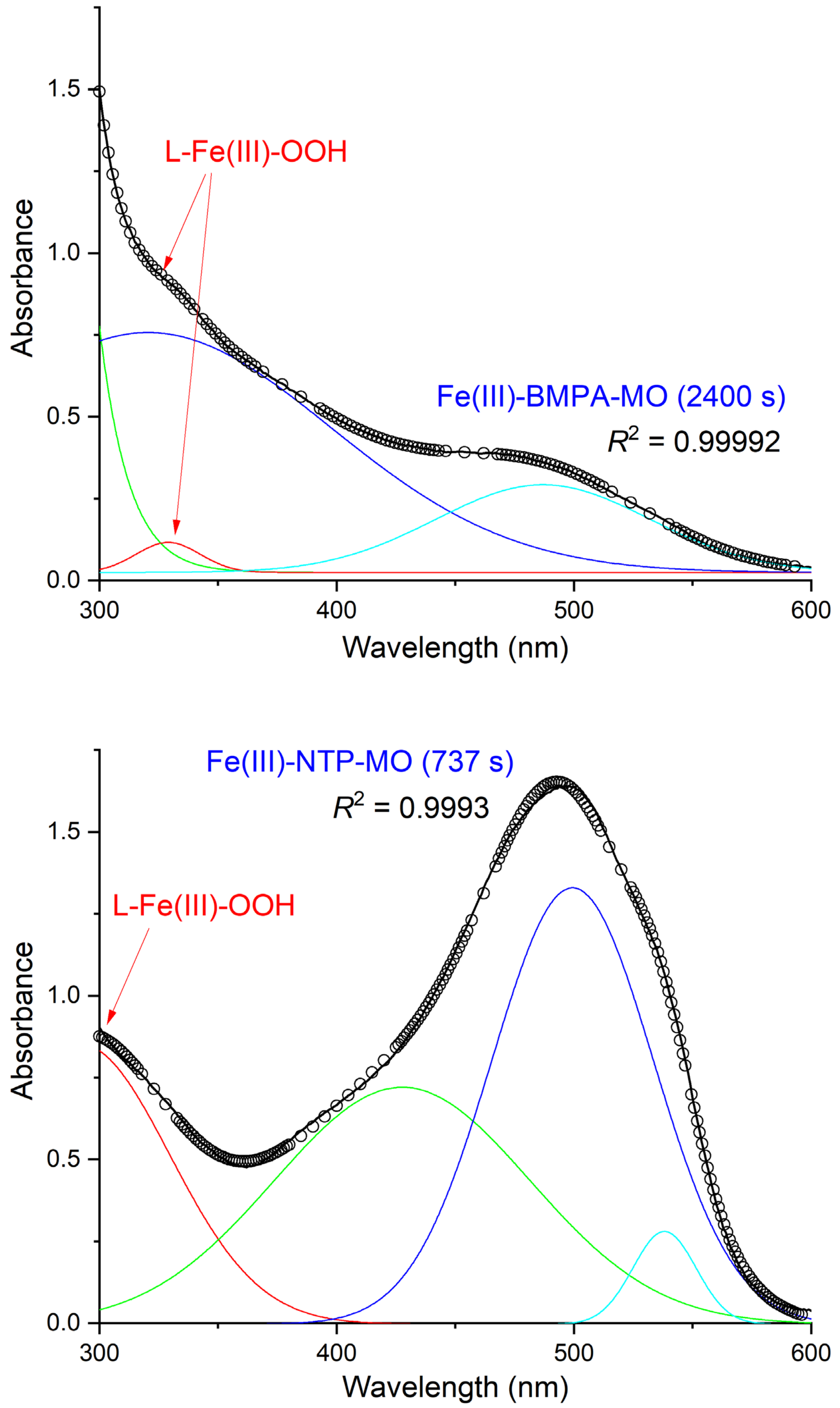

Figure S6. Spectra deconvolution for Fe(III)-BMPA-MO (up; 2400 seconds) and Fe(III)-NTP-MO (down; 737 seconds) systems. The shoulder around 320 nm, associated with the species L-Fe-OOH, is indicated with arrows. The spectra were selected choosing the time at which the absorbance of the shoulder was maximum.

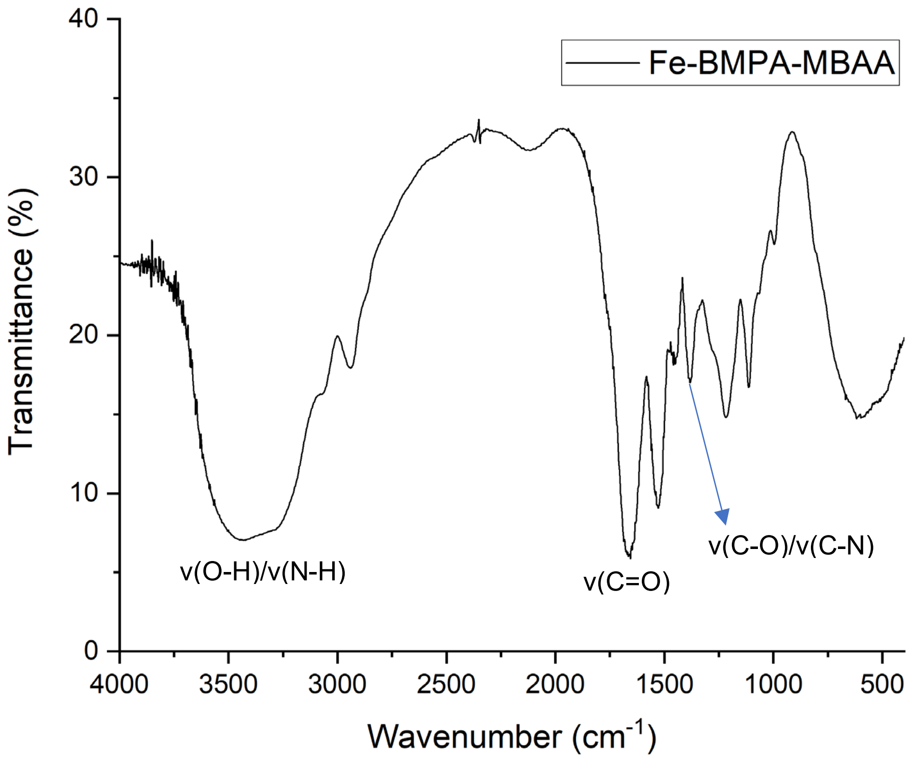

Figure S7. Infrared spectrum of Fe-BMPA-MBAA MIP.

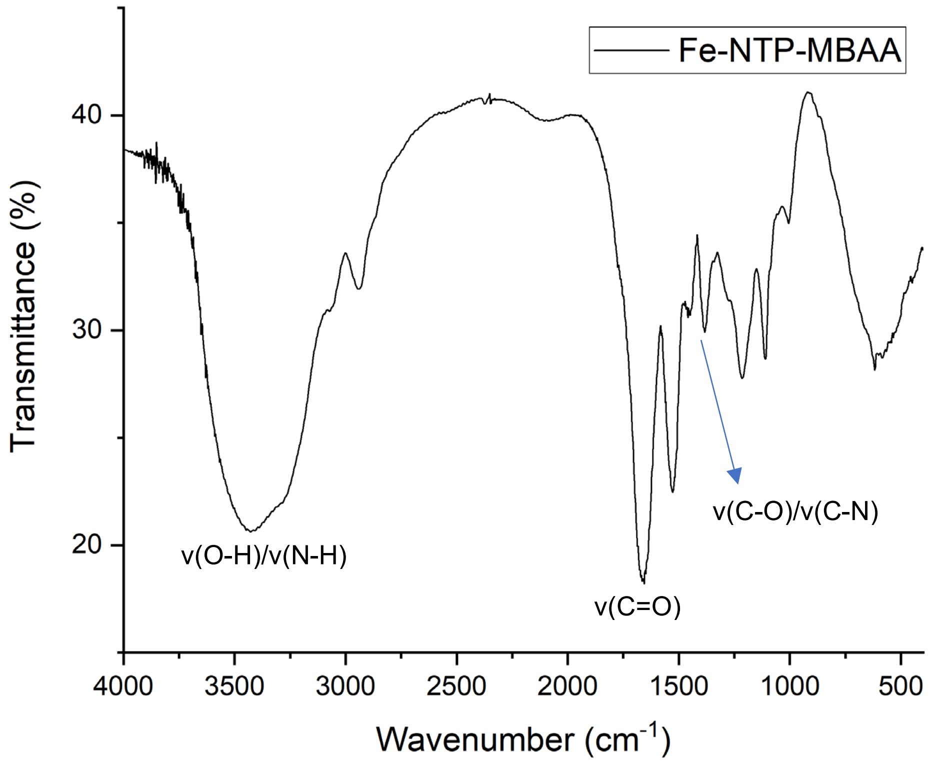

Figure S8. Infrared spectrum of Fe-NTP-MBAA MIP.

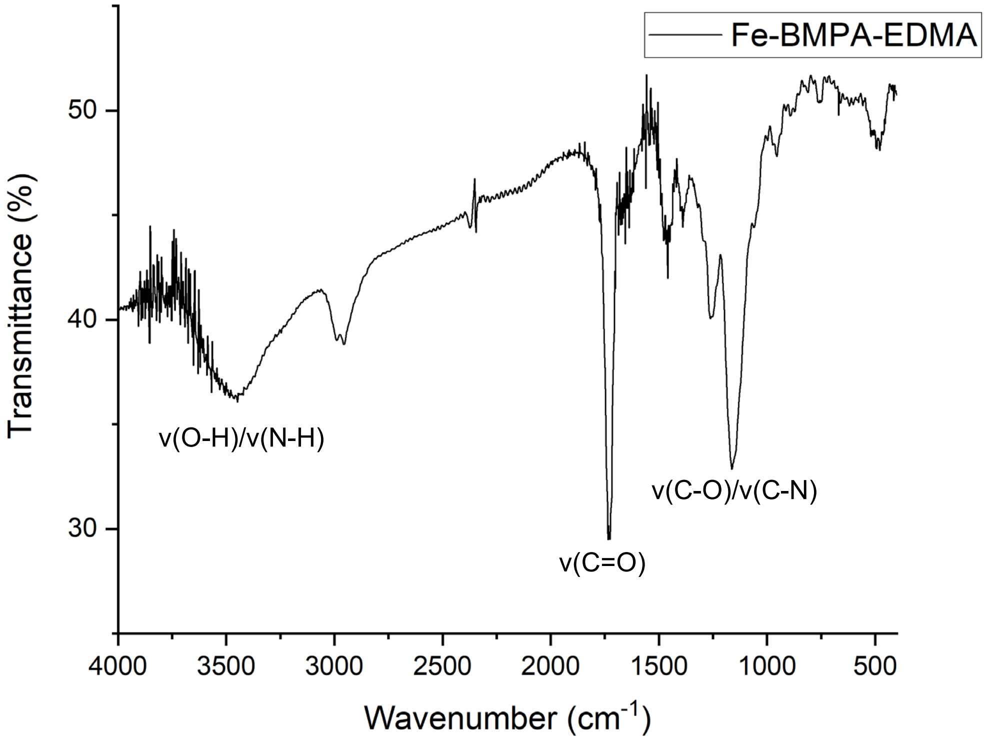

Figure S9. Infrared spectrum of Fe-BMPA-EDMA MIP.

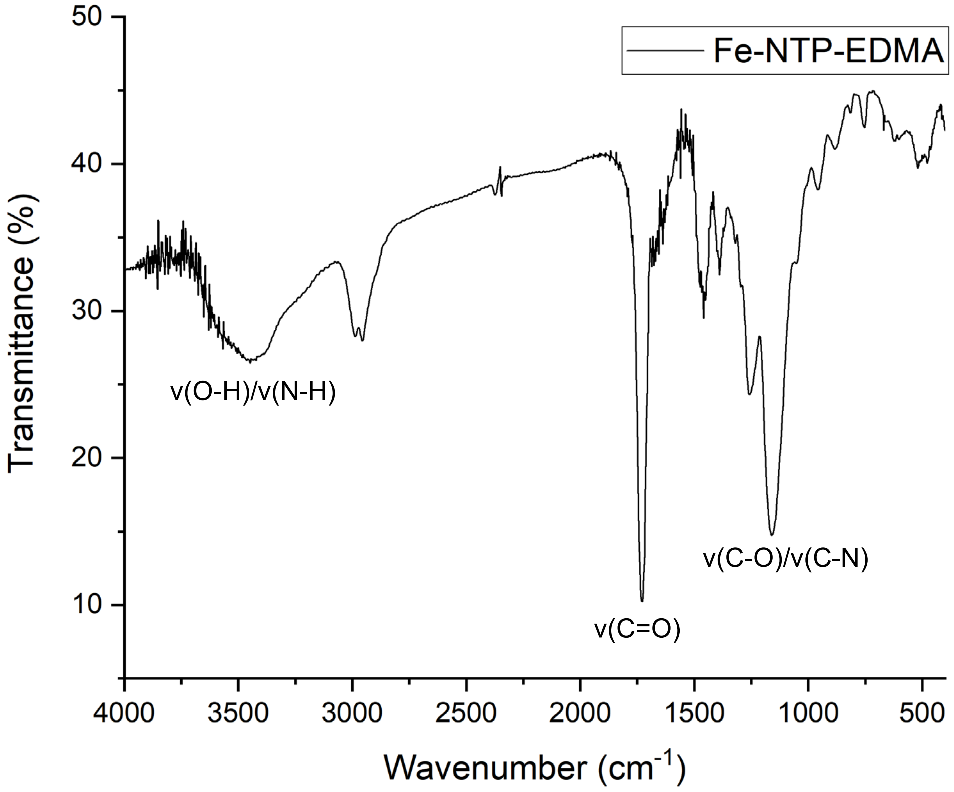

Figure S10. Infrared spectrum of Fe-NTP-EDMA MIP.

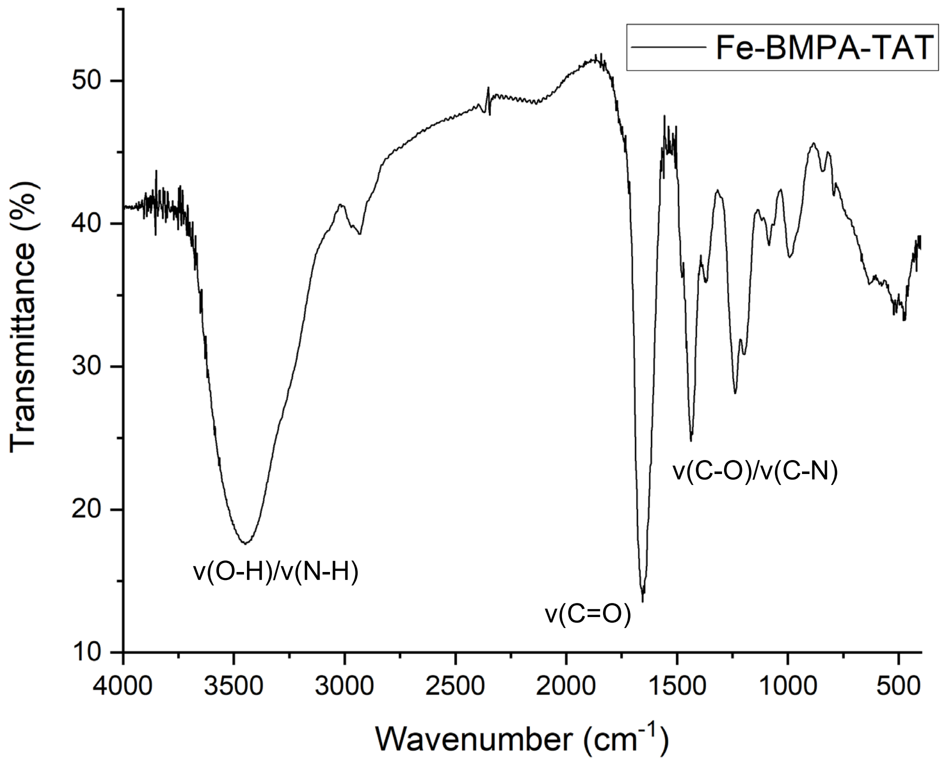

Figure S11. Infrared spectrum of Fe-BMPA-TAT MIP.

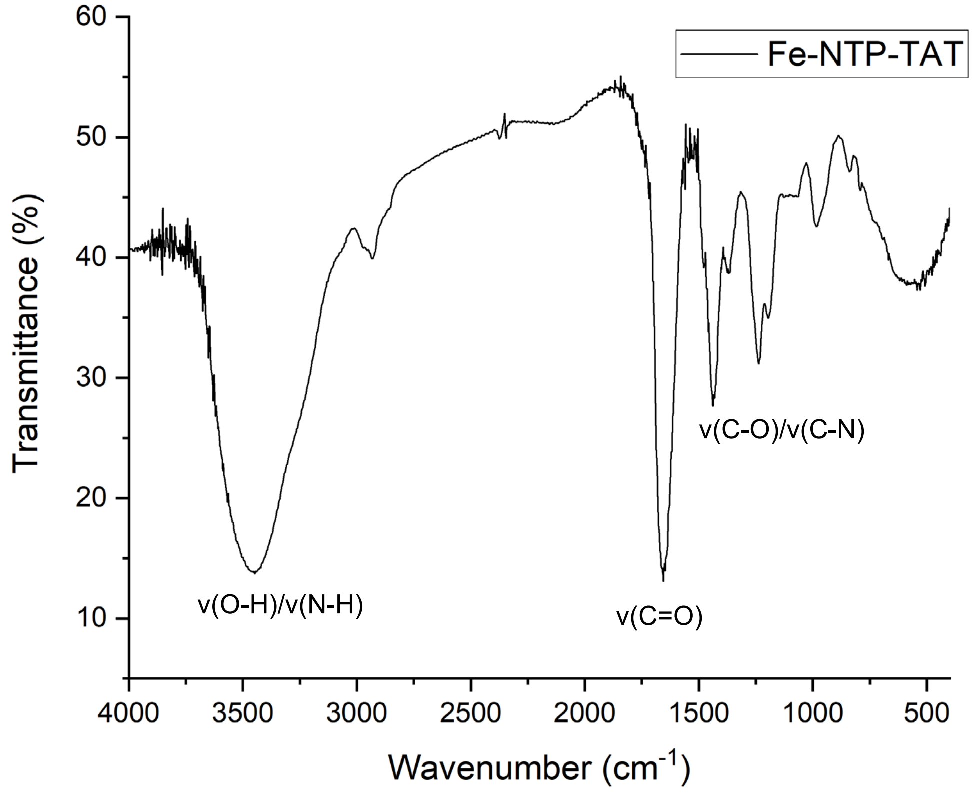

Figure S12. Infrared spectrum of Fe-NTP-TAT MIP.

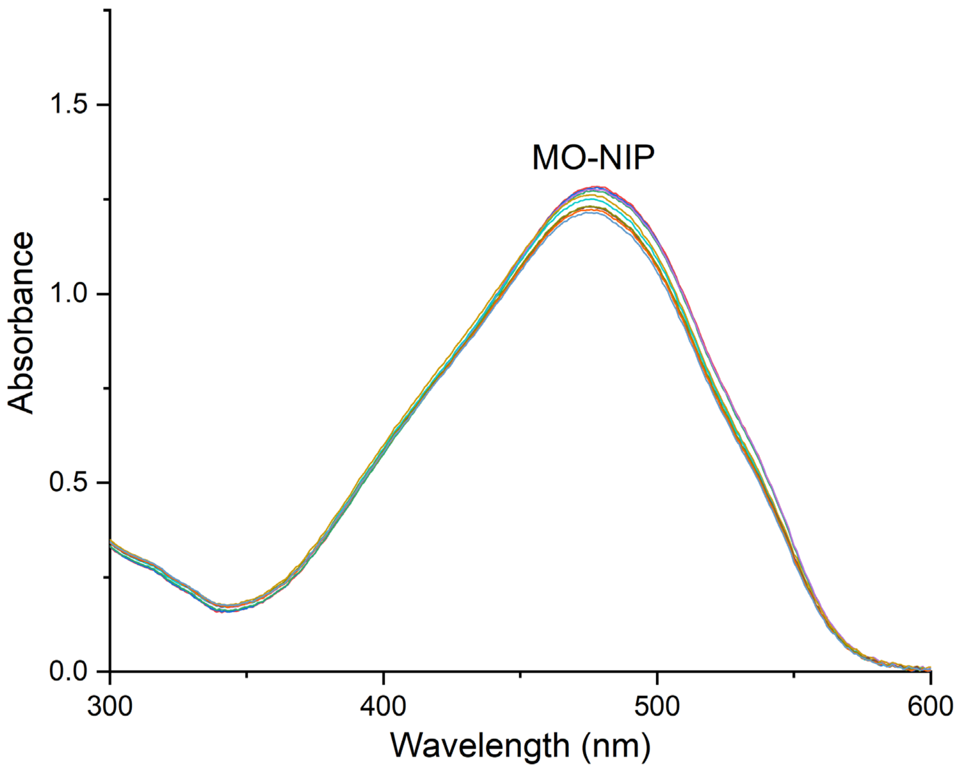

Figure S13. Catalytic performance of the MBAA NIP towards the oxidative degradation of MO in the presence of H_2O_2. Conditions: T = 20.0 °C; 3 hours; [MO] = 6 x 10^{-5} mol L^{-1}, NIP mass = 40 mg, [H_2O_2] = 2.9 x 10^{-3} mol L^{-1}.

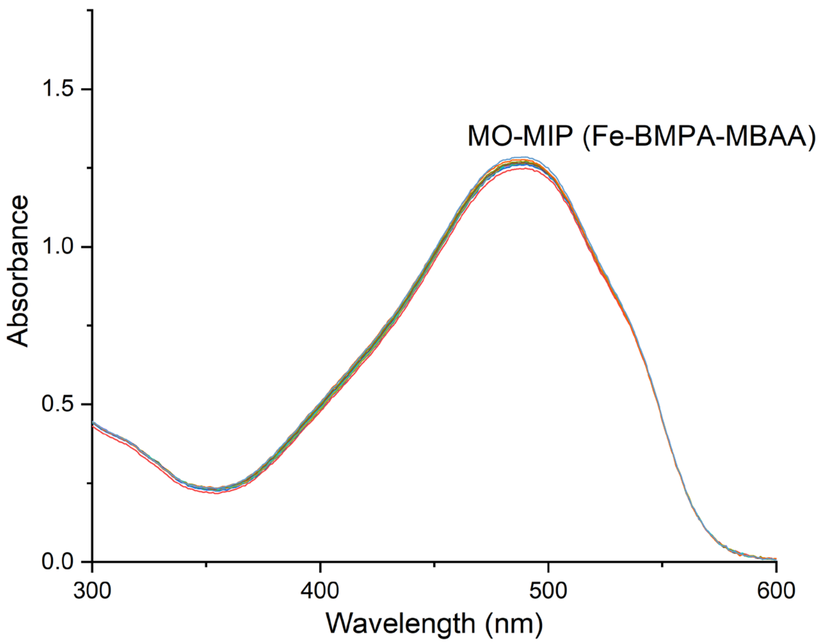

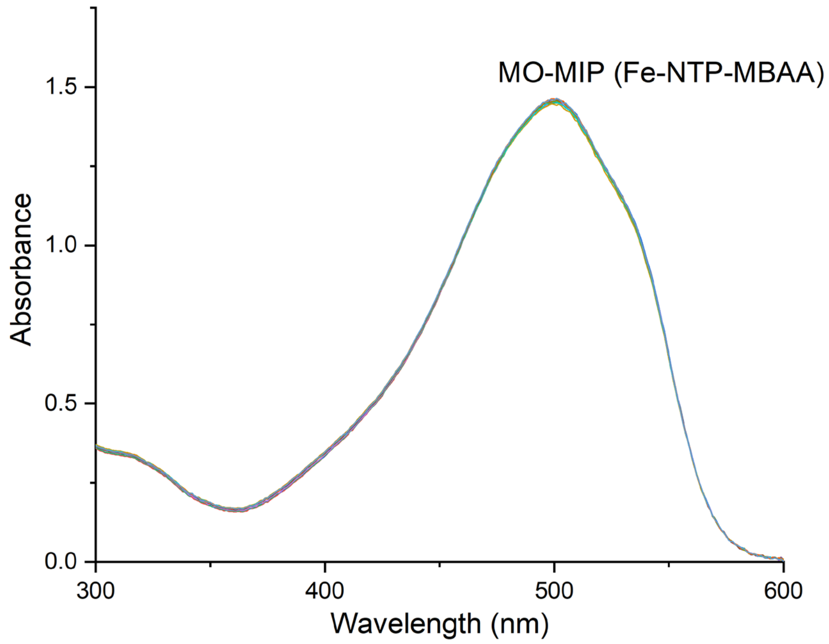

Figure S14. Catalytic performance of the Fe(III) complex imprinted polymers towards the oxidative degradation of MO **in the absence** of H_2O_2. Up: Fe(III)-BMPA-MBAA. Down: Fe(III)-NTP-MBAA. Conditions: T = 20.0 °C; 3 hours; [MO] = 6 x 10^{-5} mol L^{-1}, MIP mass = 40 mg.

Table S1. Fitting of the kinetic models.[a]

Catalyst	Goodness of fit (R^2)			
	MO degradation			Intermediate L-Fe(III)-OOH
	First-order	Second-order	Carvalho *et al.*[1]	Carvalho *et al.*[1]
	$A = A_0 e^{-kt}$	$\frac{1}{[MO]} = \frac{1}{[MO]_0} + kt$	$A = ae^{-k_1 t} + be^{-k_2 t}$	$A = a(1 - e^{-k_3 t}) + be^{-k_4 t}$
Fe(III)	0,99	0,97	0,99	0.99
Fe–BMPA	0,69	0,91	0,99	0.98
Fe–TPA	0,09	0,07	0,96	0.99
Fe–NTP	0,98	0,89	0,98	0.98
Fe–NTA	0,99	0,98	0,99	--- [b]

[a] A = MO maximum absorbance. [b] The absorbance variation at 320 nm is negligible.

Table S2. Most important infrared bands of the Fe(III) complex imprinted polymers. The assignment of the IR bands was based on previous reports.[2-6]

Polymeric matrix	Template	Wavenumber (cm^{-1})			
		ν(O-H)/ν(N-H)	ν(C=O)	ν(C=C)	ν(C-O)/ν(C-N)
MIP-MBAA	Fe-BMPA	3100-3665	1668	-	1293/1385
	Fe-NTP	3100-3660	1662	-	1301/1385
MIP-EDMA	Fe-BMPA	3300-3650	1732	-	1157/1257/1297/1393
	Fe-NTP	3300-3650	1732	-	1157/1253/1297/1393
MIP-TAT	Fe-BMPA	3130-3700	1662	-	1301/1401-1470
	Fe-NTP	3150-3700	1666	-	1401-1477/1566
Polymerization reagents					
Methacrylamide		3386/3194	1667	1608	1408
MBAA		3305	1650	1620	1301
EDMA		-	1723	1639	1154/1247
TAT		-	1650	1636	1417/1449/1563

Table S3. MO degradation for the MIPs and NIPs tested.

Crosslinking agent	MIPs		NIP
	Fe-BMPA	Fe-NTP	
MBAA	95.7%	98.7%	5.3%
EDMA	28.4%	41.8%	2.6%
TAT	23.5%	7.6%	2.3%

References

1. Carvalho, S. S. F.; Carvalho, N. M. F., Degradation of organic dyes by water soluble iron(III) mononuclear complexes from bis-(2-pyridylmethyl)amine NNN-derivative ligands. *Inorg. Chem. Commun.* **2019,** *108*, 107507.
2. Eisenreich, N.; Rohe, T., Infrared Spectroscopy in Analysis of Plastics Recycling. In *Encyclopedia of Analytical Chemistry*, 2006.
3. Reddy, B. V.; Rao, G., Vibrational spectra and modified valence force field for N,N '-methylenebisacrylamide. *Indian Journal of Pure & Applied Physics* **2008,** *46*, 611-616.
4. Soledad-Rodríguez, B., Caracterización de un Polímero de Impronta Molecular para la ampicilina por NIR. Comparación entre NIR y FT-IR. **2018,** *21*, 15-27.
5. Yusof, N. A.; Rahman, S. K. A. B.; Hussein, M. Z.; Ibrahim, N. A., Preparation and characterization of molecularly imprinted polymer as SPE sorbent for melamine isolation. *Polymers* **2013,** *5* (4), 1215-1228.
6. Reimschuessel, H. K.; McDevitt, N. T., Infrared Spectra of Some 1,3,5-Triazine Derivatives. *J. Am. Chem. Soc.* **1960,** *82* (14), 3756-3762.
